# Experimental Design and Optimization of Dispersion Systems in Fine and Specialty Chemical Fabrication

**DOI:** 10.3390/molecules31101617

**Published:** 2026-05-11

**Authors:** Sebastian J. Balicki, Izabela Klapiszewska, Łukasz Lamch, Marcin Bartman, Łukasz Klapiszewski, Kazimiera A. Wilk

**Affiliations:** 1 Faculty of Chemistry, Wrocław University of Science and Technology, 50370 Wroclaw, Poland; lukasz.lamch@pwr.edu.pl (Ł.L.); marcin.bartman@pwr.edu.pl (M.B.); kazimiera.wilk@pwr.edu.pl (K.A.W.); 2 Faculty of Civil and Transport Engineering, Institute of Building Engineering, Poznań University of Technology, 60965 Poznan, Poland; izabela.klapiszewska@put.poznan.pl; 3 Faculty of Chemical Technology, Institute of Chemical Technology and Engineering, Poznań University of Technology, 60965 Poznan, Poland; lukasz.klapiszewski@put.poznan.pl

**Keywords:** fine chemicals, specialty chemicals, process optimization, nanostructured materials, nanodispersions, application-oriented optimization

## Abstract

This review describes a process design concept suitable for the fine and specialty chemicals sector. Experimental design and optimization methodologies are powerful tools for developing and improving a wide range of products, processes, and engineering systems. The research articles thoroughly analyzed in this review demonstrate that, regardless of the analytical techniques employed or the specific processes used in the fabrication of fine and specialty chemicals, the systematic implementation of the Design of Experiments and Response Surface Methodology consistently enables the development of high-quality and reproducible outcomes. Across all the studies discussed, comparing newly developed or modified processes with conventional approaches, the application of statistically designed experiments and structured multivariate analysis resulted in significant improvements in key performance indicators. These include increased product yield, reduced process times, enhanced purity, and more precise control over the targeted functional properties of specialty and fine chemicals. Good examples that illustrate the above problem are three studies supported by data from our previously published work and our current research project, in which experimental design and process optimization play major roles in obtaining valuable nanostructured materials. These case studies—rational liquid-in-liquid nanodispersions (ND) for ecological graffiti-coating detergents, solid-in-solid nanodispersions for functionalized sustainable cementitious composites, and solid-in-liquid multicharge cationic surfactant-capped silver nanoparticles (AgNPs)—are deliberately selected to illustrate how the same systematic design and optimization principles can be applied across distinct types of dispersed systems. Together, they demonstrate a coherent methodological progression from formulation to functional material development, highlighting the versatility of this approach across different material states and application domains. The findings of this review provide a solid foundation for the optimized manufacture of novel custom-designed nanoproducts.

## 1. Introduction

The chemical manufacturing industry consists of three major segments: commodity chemicals (CCs), which are intended for mass-market applications, and fine chemicals (FCs) and specialty chemicals (SCs), whose small-scale production requires careful selection of optimal unit processes and/or optimization of commercial formulations [1,2,3,4,5,6].

Commodity chemicals are standardized substances manufactured in specialized facilities for a wide range of applications. Their prices generally below $1/kg—are cyclical and fully transparent in the market. CCs typically include basic organic chemicals (e.g., ethylene and propylene) and inorganic chemicals (e.g., soda ash and sulfuric acid). They are characterized by high production volumes and low costs. Moreover, they are mass-produced in continuous processes using the best available techniques in a given industry to meet the global demand. Finally, commodity chemicals are valued for their versatility as raw materials in downstream processing, enabling the production of a broad spectrum of products, including plastics, fertilizers, and pharmaceuticals [2,3,4].

Fine chemicals are high-quality compounds with high purity levels (typically >99%) that fulfill rigorous quality control and regulatory compliance requirements. They are marketed at prices exceeding $10 per kilogram and are produced in small batches (up to 1000 metric tons annually) through complex processes in multipurpose and multiproduct plants [2,5,6,7]. Their fabrication often requires advanced technologies and specialized analytical instrumentation and techniques, such as high-performance liquid chromatography and mass spectrometry, to ensure that the desired purity and quality standards are met. Their fine quality makes them suitable for use as active pharmaceutical ingredients (API), diagnostic agents, biosurfactants, specialty polymeric additives, biocides, and catalysts [6,8,9,10,11,12]. Their versatility and purity make them essential components of many new efficacious drugs and advanced materials. Additionally, the decreasing availability of fossil fuel resources has led to growing interest in the production of FCs using renewable energy sources. The potential reserve of biomass, including wood, sugarcane, corn, algae, and vegetable oils, is both abundant and renewable as a feedstock [2,13,14,15,16,17]. Also notable are secondary groups of these compounds comprising organic intermediates, building blocks, cosmetic ingredients, and components of reaction media [18,19,20].

Fine chemicals are generally produced within the commodity chemical segment using cost-effective manufacturing processes. They can be blended to obtain various specialty chemicals (also referred to as performance or effect chemicals), which are manufactured in large quantities and exhibit unique application-specific functionalities [3,21,22]. SCs possess specific properties that are indispensable for dedicated applications, such as improved adsorption, sorption, and adhesion; better catalytic activity; enhanced UV (ultraviolet) protection; better durability; increased resistance to light, heat, or environment; excellent antimicrobial activity; better cytotoxic ability; enhanced anticorrosive action; and improved biodegradability. Consequently, FCs and SCs are applied in a variety of industries [2], including pharmaceutical and cosmetic segments [6,11], household and detergent industries [11], specialty polymers [15], agriculture [14], the food industry, biotechnology [23,24], aerospace, automotive [13], electronics, and construction, as well as analytical and research laboratories [2,6,11,13,14,15,23,24].

Strategic planning and optimization approaches in the FCs and SCs sectors are essential for selecting the optimal input parameters that improve production processes and ensure that the resulting products exhibit the intended functional characteristics and appropriate quality levels. Experimental design (for a full description, see paragraph 2) is a well-established concept for optimizing various unit operations and production processes. However, its application in the field of fine and specialty chemicals remains relatively infrequent in the scientific literature, even though its implementation at the industrial level has been growing rapidly. The main aim of the present contribution is to indicate the most challenging aspects of experimental and modeling techniques used to identify the conditions that lead to optimal process performance, fulfillment of quality control protocols, and improved targeted functionalities of FCs and SCs. To strengthen the conceptual coherence of this contribution, the three selected case studies —graffiti-removal nanoemulsions, cationic surfactant-capped silver nanoparticles, and nanodispersed functional materials for cementitious composites—were deliberately chosen to represent three distinct classes of nanodispersed systems (liquid-in-liquid, solid-in-liquid, and solid-in-solid, respectively). Although these applications belong to different industrial domains, they collectively demonstrate how the same DoE/RSM-based (Design of Experiments, Response Surface Methodology) methodology can be systematically applied across systems with different physical states, functionalities, and formulation complexities. Their juxtaposition highlights the versatility of statistical design in solving optimization problems, ranging from formulation robustness and stability through fine-tuning of reaction and synthesis parameters to the rational design of high-performance composite materials. Together, these examples provide a coherent methodological continuum, illustrating the fundamental principles of DoE/RSM and showing how they enable efficient, knowledge-based development of specialty and fine chemical products.

Thus, the present review introduces the following issues: (i) a phenomenological description of process design suitable for this valuable sector, as well as three examples of innovative applications, such as (ii) the creation of rational and environmentally friendly water-in-oil nanoemulsions as graffiti-coating eco-remover—designed for sensitive surfaces, such as plastics, ceramics, or painted metal; (iii) optimization of unit synthetic processes of multicharge cationic surfactant-capped silver nanoparticles, colloidally stable over a long period of time; and (iv) composition optimization of nanodispersions of functional materials in sustainable cementitious composites, the latter characterized by better microstructure compaction, improved mechanical properties, and good antimicrobial function. The reported examples provide guidance for the fabrication of future customized forms of the above-mentioned specialty nanoproducts.

## 2. Concept of Experimental Design

**Figure 1:**
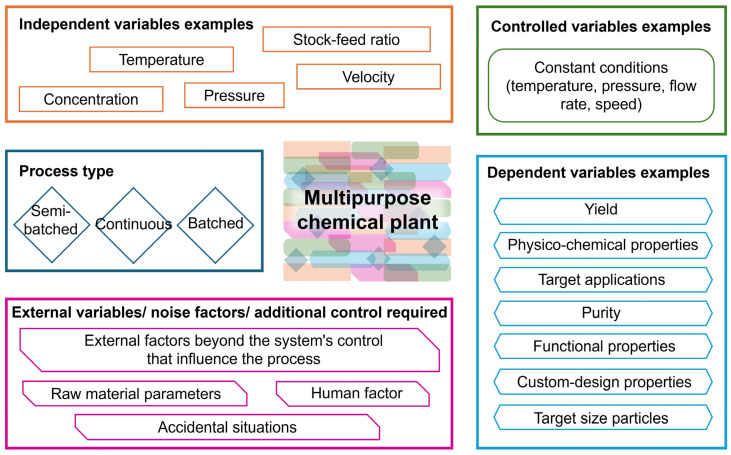
Possible statistical data and various variables to be considered by manufacturers in multipurpose chemical plants in order to implement the Design of Experiments and Quality-by-Design concepts.

The structured and methodical approach to designing experiments, known as Design of Experiments, represents the most effective strategy for solution development, as it facilitates an understanding of the cause-and-effect relationships within various interdisciplinary processes [25,26]. By employing strategic planning and careful consideration, chemical engineers can implement the DoE technique to systematically collect data, which can subsequently be applied to the creation of innovative and high-quality products (see Figure 1). This method is most effective in determining the most efficient techniques for manufacturing commercial goods of very high quality from a variety of materials. This approach is closely related to Quality-by-Design (QbD) [24].

**Figure 2:**
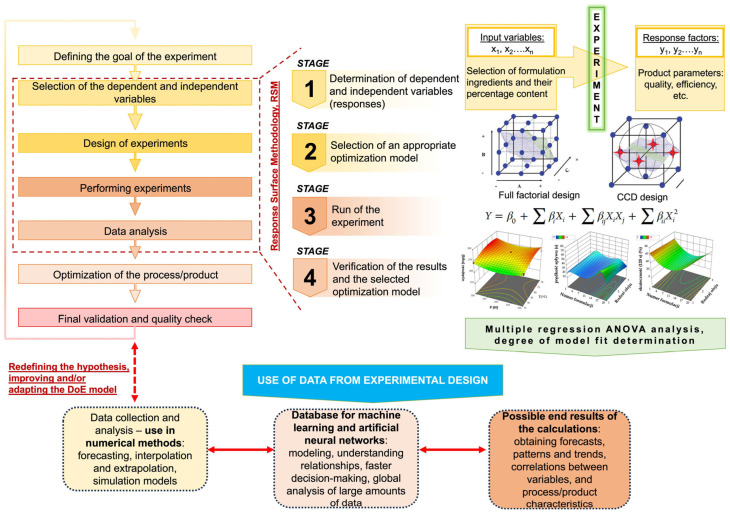
Typical procedure in the implementation of Design of Experiments and Quality-by-Design approaches.

Manufacturers of innovative formulations and various specialty chemicals in the domains of building materials, cosmetics, detergents, and various fine chemicals—including specialized surfactants, nanocarriers for drug delivery systems, novel catalyst systems, and bioactive compounds—bear the main responsibility for providing products of extraordinary quality to consumers [11,12,16,23,24,27], frequently using a multipurpose chemical plant. As the QbD approach demands, this work must be carried out with a thorough awareness of all the elements and stages involved in the production of the intended result. The concept of Quality-by-Design is that quality measures should be integrated into each stage of production (see Figure 2), not just the final stage (quality control), to ensure that the outputs are consistently of high quality. Techniques for planning and designing experiments and statistical data analyses are appropriate instruments for implementing this concept in practice [28,29].

The use of methods for experimental design and optimization of unit operations is becoming increasingly popular in the field of chemical engineering [30] and the production of specialty products and fine chemicals. Thanks to modern computational models, process engineers can successfully implement experiment planning at an early stage of research and development work and optimize the entire technological processes, achieving the assumed goals in a much shorter time [7,31].

By examining the interactions among multiple factors, Response Surface Methodology can identify the optimal parameters that yield the most favorable outcomes. The optimization process is essential for enhancing the efficiency and effectiveness of experimental designs and applications. RSM techniques frequently enable the implementation of various mathematical models that evaluate the correlations between independent and dependent variables. The study of such relationships can result in the possible optimization of unit operations, the manufacture of specialty and fine chemicals, or the development of various nanodispersion formulations. RSM, by employing computational and numerical methods, enables the definition of an algorithm with methodical procedures for conducting a defined number of experiments, ensuring that the gathered experimental data is in accordance with the data calculated through the DoE models. The trade-off among a broad spectrum of potential optimal solutions can be analyzed and evaluated using a variety of derivatives of RSM methodologies rooted in distinct computational and statistical frameworks. This leads to the selection of the optimal solution to the engineering problem (see Figure 2) [32,33,34]. The aforementioned frameworks can be divided into three main groups: DoE, conventional numerical methods such as statistical analysis, and computational methods using artificial or neural networks. Examples of these approaches are briefly described in Table 1.

The unit and/or process operation, as well as a particular fine chemical that needs to be produced, are the primary focus of the process optimizations described in Table 1. The implementation of these improvements is necessary to enhance efficiency and reduce costs. Producers can significantly improve both their output and sustainability by focusing on specific procedures and chemicals. By implementing these improvements, the chemical industry will become more environmentally friendly and, at the same time, more profitable. Organizations that use these strategies should see an increase in their overall market competitiveness as a result of their implementation. For example, it can be used to optimize fine or specialty chemicals, such as the extraction of gallic acid and lycopene described by S. Pandey and S. Kumar [35] and S. Rahimi and M. Mikani [36]. In contrast, chemical and physical processes, such as synthetic routes, revalorization, and various extraction methods, can also be optimized [37,38,39]. Finally, the experimental studies presented in Table 1 implemented various approaches for optimization, including single-step optimization, design of experiments combined with further optimization using two different mathematical models, and multi-step and multivariate optimization designs. These distinct methodologies not only enhance the efficiency of extraction and synthesis procedures but also facilitate our comprehension of the underlying processes. These methods are likely to become more cost-effective and useful in a variety of disciplines as researchers continue to refine them, resulting in improved yields and purities. This modification of optimization methods will likely result in the development of novel solutions in fields such as pharmaceuticals and materials science. Consequently, the subsequent iteration of technological advancement will be significantly influenced by the integration of advanced modeling and experimental design [34]. Consequently, the Design of Experiments methodology, as a foundational element of numerical and computational modeling, becomes an essential component of process and product quality design in the fine and specialty chemicals sector, particularly when integrated with artificial neural network-assisted optimization. This integration enhances predictive accuracy and significantly reduces the time required for experimentation and development. As a result, organizations can achieve more efficient production processes while maintaining high-quality standards in their products. The scientific and professional literature frequently describes DoE-based numerical and computational methods for optimizing dispersion system formation (more than 4300 research papers indexed in the Web of Science, Clarivate database, over the past 10 years; www.webofscience.com, accessed 29 January 2026 35C). However, the emphasis on nanodispersions in this review highlights their unique characteristics and importance in the context of dispersion formation. In the subsequent sections, we provide an in-depth exploration of these systems and their optimization processes.

**Table 1:** Comparison of different approaches, in the design of experiments, i.e., DoE, numerical, and artificial neural network in the manufacture process of fine chemicals (FCs) and specialty chemicals (SCs), followed by differentiation in dependent and independent variables.

Chemical or Physical Process	Details of the Experiment	Variables	Applied Computational Method	Ref.
Reactive extraction of gallic acid from aqueous solution with *tri*-n-octylamine in oleyl alcohol, a recovery from waste waters	The optimal conditions for extraction of HGA were CHGA,o = 0.0588 4 mol/L, C¯TOA,o = 0.2762 mol/L, pH = 2.0, and temperature T = 25.0 °C. 90.09% extraction yield is predicted by the RSM model, validated one 87.17%.	Independent: initial gallic acid concentration (A); *tri*-n-octylamine concentration (B); pH of aqueous phase (C); temperature (D). Dependent: extraction yield (%)	Statistical analysis and optimization by RSM-Rotatable Central Composite Design (RSM-RCCD) model, followed by multiple regression analysis	[36]
Lycopene green ultrasound-assisted extraction using edible oil, a processing of tomato wastes	UAE reached its maximum yield (lycopene of 91.49 mg/100 g) in 10 min, while conventional solvent extraction using hexane and mixture (hexane: acetone: methanol at 2:1:1 *v*/*v*) gave lycopene concentrations of 63.66 mg/100 g and 74.89 mg/100 g after 1 h, respectively.	Independent: Ratio of dried tomato waste to oil (A); extraction time (B); ultrasonic intensity (C). Dependent: total lycopene yield (mg/g)	Statistical analysis and optimization by RSM-Central Composite Design (RSM-CCD) model, followed by multiple regression analysis	[37]
Microwave-assisted extraction of polysaccharides from the marshmallow roots	The maximum MRPs extracted experimentally were found to be 14.51 ± 0.06%, after 26 min, using microwaves. Conventional and ultrasound extraction gave 10.96 and 12.15%, after 12.01 h and 36.86 min of process time, respectively.	Independent: microwave power (A); time (B); temperature (C). Dependent: extraction yield (%)	Statistical analysis and optimization by RSM-Central Composite Rotatable Design (RSM-CCRD), followed by multiple regression analysis	[38]
Microwave-assisted extraction of cocoa bean shell waste as a potential antioxidant source	Optimal MAE conditions were determined as 5 min, pH 12, 97 °C, and S/L 0.04 g/mL, showing a better outcome than conventional solvent extraction performed at 90 min and 100 °C and 0.045 g/mL ratio.	Independent: time (A); pH (B); temperature (C); solid to liquid ratio (D). Dependent: yield (%); uronic acid content (mg GlcA/g); total phenolic content (mg GAE/g); antioxidant activity (mg TE/g)	Statistical analysis and optimization by RSM-Box-Behnken Design (RSM-BBD), followed by multiple regression analysis	[40]
Extraction of capsaicin from *Capsicum annum* L.	The higher CAP content (0.0163 mg/g DW) was recorded with the following conditions: 90 °C drying temperature, 54 g/L concentration, and 48.75 min of extraction with acetonitrile. ANN prediction was more accurate than RSM and Simulink with a higher coefficient of determination (*R*^2^) (0.9901 vs. 0.9602 and 0.9607, respectively).	Independent: drying temperature (A); sample to solvent ratio (B); extraction time (C). Dependent: capsaicin yield (mg/g); dihydrocapsaicin yield (mg/g); total capsaicinoids yield (mg/g)	Statistical analysis and optimization by RSM-I-optimal Randomized (Custom) Design (RSM-I-optimal), followed by multiple regression analysis; Artificial Neural Network (ANN), A feed-forward model was created utilizing a hyperbolic tangent sigmoid transfer function in the hidden layer, along with a linear transfer function in the output layer. The network underwent training utilizing the Levenberg–Marquardt back-propagation algorithm; Simulink, a MATLAB mathematical software extension for modeling and simulation of systems. The numerical model was developed based on equations resulting from multiple regression analysis done by RSM	[41]
Revalorization of waste cooking oil by esterification and deacidification for biodiesel production	The optimum conditions of the ratio of methanol to oil ratio (8), H_2_SO_4_ catalysis concentration (5 wt%), reaction temperature (60 °C), and reaction time (108 min) were obtained and an acid value of 0.42 was achieved.	Independent: reaction time (A); temperature (B); catalyst concentration (C); molar ratio of methanol to oil (D). Dependent: acid values (mg KOH/g)	Statistical analysis and optimization by RSM-Box-Behnken Design (RSM-BBD), followed by multiple regression analysis	[39]
Optimization of the synthesis conditions of TiO_2_/biochar composites	Central-Hybrid Experimental Design revealed that biochar produced at 280 °C with 4.1% *v*/*v* oxygen and a TiO_2_ /biochar weight ratio of 1.5 yielded the best results.	Independent: pyrolysis temperature (A); oxygen content in pyrolysis (B); TiO_2_ to biochar ratio (C); calcination temperature (D). Dependent: degradation percentage of polymeric matrix	Numerical optimization by 416B-type central-hybrid experimental design, followed by statistical analysis	[30]
Synthesis for the 4-Pyridone Intermediate of Baloxavir Marboxil	The optimized process was successfully scaled up to 135 g in the laboratory, yielding the monohydrate form of the compound with a purity of 98.3% and an overall yield improved from 78.6% to 85.1%.	Independent: screening: addition time of *tert*-butyl carbazate (A1); triethylamine (TEA) catalyst equivalent (B1); temperature (C1); solvent volume (D1); optimization: triethylamine (TEA) catalyst equivalent (A2); temperature (B2); solvent volume (C2). Dependent: both screening and optimization: content of product; combined content of degradation impurities; content of hydrazone impurities—analyzed through HPLC.	Statistical analysis and optimization by combining stage one: Definitive Screen Design (DSD), followed by the second stage: RSM-Central Composite Design (RSM-CCD), followed by multiple regression analysis	[42]
Optimization of Eudragit RS100 nanocapsule formulation for encapsulating perillyl alcohol and temozolomide	The optimized nanocapsules demonstrated a mean diameter of 253 ± 52 nm and a polydispersity index of 0.145 ± 0.037. Formulation achieved an average particle size under 300 nm, a PDI indicating homogeneity, and a stable zeta potential, which is favorable for intranasal delivery	Independent: Eudragit RS100 concentration (A); perillyl alcohol concentration (B); drip rate of drops (C); organic to aqueous phase ratio (D). Dependent: average diameter of particles; polydispersity index; zeta potential; encapsulation efficiency	Statistical analysis and optimization by RSM-Factorial Design (RSM-FD), followed by multiple regression analysis	[23]
Synthesis of alginate hydrogel functionalized by cationic surfactant for efficient perfluorooctanoic acid adsorption	The optimal hydrogel exhibited an average PFOA removal efficiency of 94.8 ± 2.1% at a 50 mg/L PFOA. The experimental data for the hydrogel closely align with the pseudo-second-order rate kinetic model, with a maximum possible adsorption capacity of 382.1 mg/g	Independent: cetyltrimethylammonium bromide (CTABr) concentration (A); calcium concentration (B); sodium alginate concentration (C). Dependent: perfluorooctanoic acid removal (%)	Statistical analysis and optimization by RSM-I-optimal Randomized (Custom) Design (RSM-I-optimal), followed by multiple regression analysis	[11]
Process development and optimization of apalutamide synthesis, aided by the Design of Experiments (DoE)	The overall process yield for apalutamide reached 70% with an HPLC purity of 99.97%, after implementation of DoE/RSM optimization.	Multi-step organic synthesis of apalutamide. Step 1, independent: temperature (A); time (B); 1-aminocyclobutane-1-carboxylic acid amount (C); K_2_CO_3_ amount (D); CuI amount (E); H_2_O amount (F). Dependent: impurity concentrations; quality of semi-product 1-((3-fluoro-4-(methylcarbamoyl)phenyl)amino) cyclobutane-1-carboxylic acid. Step 2, independent: temperature (A); 1′-carbonyldiimidazole (CDI) amount (B); intermediate 5 amount (C); time (D); 1,8-diazabicyclo [5.4.0]undec-7-ene (DBU) amount. Dependent: impurity concentrations; quality of semi-product 4-((1-((6-cyano-5-(trifluoromethyl)pyridin-3-yl) carbamoyl)cyclobutyl)amino)-2-fluoro-N-methylbenzamide. Step 3, independent: temperature (A); time (B); intermediate 15 amount (C); 4-dimethylaminopyridine (DMAP) amount (D); N,N-dimethylacetamide (DMAc) solvent amount (E). Dependent: impurity concentrations; quality of final product apalutamide 1	Definitive Screening Designs and Custom Designs were employed to sieve and optimize the significant factors to establish the experimental ranges for each reaction step	[43]
Biohydrogen gas synthesis from food waste hydrolysate	The modified Gompertz model revealed a maximum bioH_2_ production rate of 185.34 mL/L·h for ANN-GA conditions as compared to 153.74 mL/L·h for RSM-CCD predicted conditions.	Independent: total reducing sugars (TRS) concentration (A); pH (B); temperature (C). Dependent: cumulative hydrogen production (CHP)	The physicochemical parameters for bioH_2_ production were optimized using the Response Surface Methodology (RSM) based on 5-level-3-factor Central Composite Design (CCD), followed by ANOVA and multiple regression analysis. Results from CCD served as data set for an Artificial Neural Network (ANN) in decision-making for nonlinear systems	[44]
Bovine serum albumin nanoparticles	Full factorial design experiments systematically evaluated the effects of PGPR amount (5–20 wt%), TDI amount (50–100 mg), and co-emulsifier (PG3DIS) use in formulations.	Independent: Emulsifier (PGPR) amount (A); Crosslinker (TDI) amount (B); Co-emulsifier Ratio (PG3DIS:PGPR) (C). Dependent: mean particle size, mean polydispersity index (PDI)	The OFAT (one factor at a time) approach based on 2-level-3-factor Full Factorial Design, followed by reduced ANOVA and multiple regression analysis.	[45]
Dispersion of calcium phosphate nanoparticles for cellular studies	Optimization using a second-order CCD yielded a set of quadratic regression equations that were used to predict the hydrodynamic size or zeta potential of ceramic nanoparticles with high accuracy (R^2^, 0.88–0.92)	1st stage (screening), Independent: concentration (A); ethanol pre-wetting (B); BSA additive (C); sonication type (D); pH (E); dispersion medium (F). Dependent: particle size, zeta potential. 2nd stage (optimization), Independent: concentration (A); pH (B); BSA additive (C). Dependent: particle size, zeta potential.	The particle size and zeta potential of nanoparticles were optimized in two stages. In the first one, a Placket-Burman 2-level-6-factor Design allowed for the screening of variables contributing to the particle size and zeta potential. The second stage was based on a 3-level-3-factor second-order Central Composite Design (CCD) response surface methodology (RSM) followed by ANOVA and multiple regression analysis.	[46]
Production of itraconazole (ITZ) amorphous solid dispersions (ASDs) by extrusion process	Validation studies confirmed optimal process robustness across multiple days, with stable in-line UV–Vis spectra and consistent product quality using 30% ITZ, 300 rpm, 150 °C, and 7 g/min	1st stage (screening), Independent: ITZ concentration (A); die temperature (B); screw speed (C); feed rate (D). Dependent: absorbance at 370 and 390 nm, 2nd stage (optimization, Independent: screw speed (A); feed rate (B). Dependent: absorbance at 370 and 390 nm, L* (function of ITZ concentration).	The parameters for the manufacture of ITZ solid dispersions were optimized in two stages. The screening stage was based on a 3-level-4-factor Fractional Factorial Design. While the optimization stage was based on a 3-level-2-factor Central Composite Design (CCD). Both stages were followed by statistical analysis employing ANOVA, main effects, and two-factor interactions modeling.	[47]

The research articles summarized in Table 1 demonstrate that, regardless of the analytical techniques employed or the specific type of process used in the fabrication of fine and specialty chemicals, the systematic application of DoE and RSM consistently enables the generation of high-quality and reproducible results. Across all studies in which newly developed or modified processes were compared with traditional or conventional approaches, the use of statistically planned experiments and structured multivariate analysis led to marked improvements in key performance indicators, such as higher product yield, shorter process times, enhanced purity, and better control of targeted functional properties. These findings collectively reinforce the value of DoE/RSM as a versatile and reliable methodology for optimizing complex formulations and production pathways in the FCs and SCs sectors.

## 3. Ecological Nanodetergents for the Removal of Graffiti—Optimization of the Method and the Formulation

Graffiti vandalism remains a widespread and costly problem in cities, places of cultural importance, and modern structures. Paints, which are typically composed of acrylic, vinyl, alkyd, or bituminous binders, create chemically stable polymer films. These coatings exhibit strong adhesion to a variety of substrates, including plastics, ceramics, tempered glass, metals, and both natural and synthetic stones. Conventional cleaning methods often employ aggressive organic solvents and mechanical cleaning procedures. As a result, using inadequate or inappropriate methods to remove graffiti can cause permanent damage to the surface. This damage may appear as dullness, discoloration, tiny scratches, or changes in the interaction of the surface with other materials [48,49,50].

The advent of nanostructured fluids (NSFs) has introduced a novel methodology for removing undesired polymer coatings. Piero Baglioni and colleagues [51] have shown that microemulsions and nanoemulsions, formulated with biodegradable surfactants, can selectively eliminate polymer coatings from artworks and historical artifacts while avoiding substrate penetration or damage. The most effective formulations offer highly precise control over wetting and dewetting phenomena [51,52,53]. Later studies confirmed that the use of nanostructured films (NSFs) in hydrogels improves selectivity and reduces damage to delicate surfaces [53,54]. These findings support the development of environmentally friendly nanodetergents. These detergents are designed to clean valuable surfaces that have been damaged by polymer coatings, including those used in graffiti removal.

Water-in-oil nanoemulsions, characterized by their adjustable physical, chemical, and surface properties, offer a promising method for graffiti removal. These novel nanoemulsions facilitate the effective removal of graffiti paint from sensitive surfaces while minimizing environmental consequences. The formulation of water-in-oil nanoemulsions illustrates the utilization of DoE and RSM, which aligns with the tenets of QbD, a methodology commonly employed in the specialty chemicals sector [6,15,28,29].

To develop stable and highly effective nanodetergents, research was conducted using a D-optimal experimental design. This approach allowed for the simultaneous evaluation of several qualitative and quantitative factors. These included the type of surfactant (alkyl polyglucosides (APG) and amino acid surfactants (AAS)), surfactant concentrations ranging from 0.05 to 0.10 mol, and the specific type of PEG-8 vegetable oil ester used: PEG-8 rapeseed oil (RO PEG-8), PEG-8 sunflower oil (SO PEG-8), and PEG-8 from used cooking oil (UCO-PEG-8). Each formulation contained 38.5 wt% PEG-8 oil ester, 45 wt% biosolvents (including ethyl lactate (EL), D-limonene (LIM), and 3-methoxy-3-methyl-1-butanol (MMB)), and 14 wt% water. This strategy is consistent with the principles of modern green chemistry—in particular, the 6R concept (Refuse, Reduce, Reuse, Recover, Recycle, Rethink)—which uses renewable raw materials, biodegradable surfactants, and environmentally friendly solvents [55].

A key technological step in the production of nanostructured fluids was the use of industrial high-pressure homogenization (HPH) to produce w/o nanoemulsions. The process of homogenization significantly affects the droplet diameter (DH), polydispersity index (PDI), and kinetic stability, as measured by the Turbiscan Stability Index (TSI). Analysis using the DoE method showed statistically significant relationships between the surfactant type, its concentration, the homogenization pressure, and the type of oil used.

The D-optimal model successfully captured the nonlinear characteristics inherent in the system’s response, specifically the pronounced interactions observed between surfactant concentration and homogenization pressure, as well as between surfactant type and oil phase composition, as detailed in Table 2. These interactions, in turn, facilitated the formation of nanoemulsions. These emulsions exhibited droplet sizes ranging from 100 to 500 nm, demonstrated low polydispersity index (PDI) values (below 0.05–0.10), and maintained stability over an extended period (with a TSI of less than 5 after 90 days). Consequently, this validated the efficacy of response surface methodology (RSM) as a suitable technique for optimizing intricate colloidal systems [56,57,58,59].

**Figure 3:**
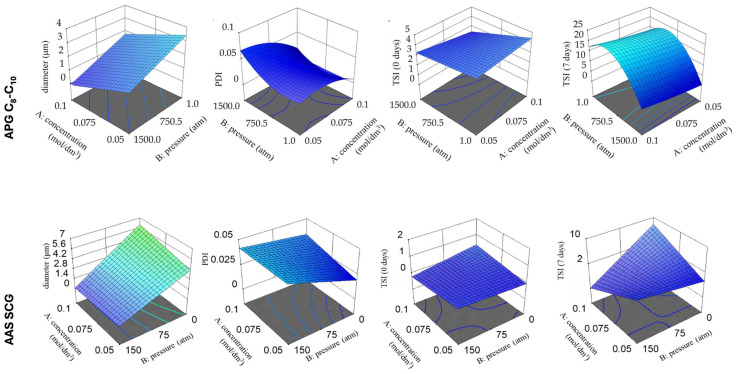
Graphical representation of the response surfaces as a function of D-Optimal RSM model for dependent variables: Y_1_ = diameter, Y_2_ = PDI, Y_3_ = TSI (0 days) and Y_4_ = TSI (7 days) vs. independent variables: alkyl polyglucoside surfactant (APG) concentration (A), HPH pressure (B) as a function of APG type: C_8_-C_10_ carbon atoms in the alkyl chain; AAS concentration (A), HPH pressure (B) as a function of AAS type: Sodium Cocoyl Glycinate (SCG)—as the optimal candidates for nanostructured fluids.

Comparative analyses showed that formulations stabilized with APG surfactants with variable alkyl chain lengths (especially APG with an alkyl chain in the C_8_–C_10_ range of carbon atoms) formed nanoemulsions with DH ≈ 0.175 μm and very low polydispersity, which favored the rapid dewetting process of polymer graffiti coatings (Figure 3). The strong interfacial activity of APG surfactants results from the combination of hydrophilic sugar heads with hydrophobic medium-length alkyl chains, which allows for the effective reduction of interfacial tension and penetration of hydrophobic paint structures [59,60,61,62,63].

Consequently, nanoemulsions stabilized with amino acid surfactants, such as Sodium Cocoyl Glutamate (SCG) or Sodium Methyl Cocoyl Taurate (SMCT), produced larger droplet diameters than those stabilized with APG but were characterized by better colloidal stability (Table 3). Compared to their cleaning properties, w/o nanoemulsions stabilized with AAS exhibited slower but more controlled action, which is particularly advantageous for highly sensitive substrates such as painted metals, polished stones, or materials of conservation importance [64,65].

The differences in the mechanisms of action of the APG and AAS surfactants were confirmed by the wettability measurements presented in Table 4. APG nanoemulsions exhibited higher spreading work (WS) and adhesion work (WA) on hydrophobic graffiti coatings, which facilitated rapid disruption and removal of the polymer layer. Consequently, systems stabilized by AAS showed lower WS values at higher surface energy, which led to gentler interactions and reduced risk of substrate damage [51,52,53,54]. This finding highlights the crucial role of surfactant structure, concentration, conditions of use, and solvent composition in balancing effectiveness with surface protection.

Standard microemulsion and gel systems used for preserving cultural heritage often have droplet sizes larger than 500 nm and show only moderate stability over time. Conversely, water-in-oil nanoemulsions are characterized by considerably smaller droplet dimensions, typically ranging from 170 to 250 nm, and substantially lower TSI values, generally below 3. Unlike gel systems, which are limited by the extent to which solvents spread, nanodetergent formulations offer a more effective and safer way to remove contaminants.

These formulations facilitate controlled activity at interfaces and selectivity across diverse surfaces. Incorporating sustainable development principles is crucial when designing formulations. The use of green solvents and renewable raw materials, such as ethyl lactate, D-limonene, and PEG-8 esters derived from vegetable oils or used cooking oils, illustrates this point. These substances align with the principles of ecological chemical product design owing to their reduced toxicity, good biodegradability, and beneficial environmental attributes [55]. Furthermore, the incorporation of surfactants, such as APG or AAS, contributes to the ecological profile of the formulation.

The practical use of DoE significantly reduces the number of experiments required. In practice, the use of Plan D-Optimal reduced the number of necessary experiments by approximately 70% compared to the single-factor approach. This allowed the identification of stable process regions, which translates into the scalability of the production method [28,29,30,31,34]. The integration of DoE and RSM allowed the identification of important formulation and process parameters, reduced experimental effort, and the production of nanoemulsions with very favorable physicochemical and functional properties. These results provide a solid methodological basis for designing the next generation of specialized cleaning preparations based on nanodispersions, contributing to sustainable, effective, and selective solutions for removing graffiti coatings from sensitive surfaces.

## 4. Approach to the Production of Cement Composites

Nanomaterials, such as metal oxide nanoparticles, continue to be the focus of applied research.

In the construction sector, they are used as additives and admixtures in cement-based composites. Among the most commonly used nano-oxides are nano-SiO_2_ [66], nano-TiO_2_ [67], nano-ZnO [68], nano-Fe_2_O_3_ [69], and nano-CuO [70]. Their introduction into the cement matrix results in a type of nanodispersion, where fine nanomaterial particles are suspended in the cement paste (three-component system) or cement mortar (four-component system). Despite the wide application of various nanomaterials in cement composites, their incorporation into cement matrices is associated with certain limitations. The most commonly observed problem is the tendency of nanomaterials to form aggregates and agglomerates. As a result, the metal oxide nanoparticles are not uniformly distributed throughout the volume of the material. These composites are characterized by local variations in strength, microstructure, and additional functionalities imparted by the admixture [71]. As research on the formation of nanodispersions of nanoparticles in cement composites progressed, it was observed that predispersing the admixture in the mixing water using a magnetic stirrer, followed by its introduction into the mixing bowl, effectively reduced the formation of aggregates and agglomerates of nanoparticles. Very good results were obtained by Klapiszewska et al. [72], who tested the effect of a superplasticizer. This addition effectively inhibited the formation of unwanted, larger particle structures. Sikora et al. [70] demonstrated the beneficial effect of using an ultrasonic bath in the initial stages of producing a dispersion of nano-oxides in mixing water. A new approach to preventing nanoparticle aggregation involves developing materials that not only contain the desired admixture for incorporation into the cement matrix but also enhance their own dispersion. To date, proposed modifications include the creation of hybrid materials containing these metal nano-oxides, such as those combined with lignin [73,74], as well as the synthesis of deep eutectic solvents containing nano-ZnO [75]. After incorporating these admixtures into cement composites, a homogeneous distribution of nano-oxides, changes in porosity, and modifications of material properties were observed.

The study of cement composites modified with different types of admixtures involves an extensive experimental process in which both the amount of newly introduced material and fixed parameters typical of cement composites, such as the water/binder and water/aggregate ratios, are studied. This results in many results that must be carefully analyzed to identify the product with the most desirable properties. The use of statistical and analytical tools is invaluable in the design of experiments and the analysis of results. The correct identification of the independent variables related to the parameters introduced into the process and the dependent variables (the responses of the system) allows an effective verification analysis and helps identify the product with the most advantageous parameters. Table 5 summarizes the studies dedicated to the application of experimental design methodology in research on cement composites, listing the materials investigated, the identified independent and dependent variables, their significance, and the methods used to present the results, including the applied calculation procedures. Among the experimental works presented, the largest group consisted of cement mortars containing metal oxide admixtures or metal oxide–biopolymer hybrid systems, as well as self-compacting mortars.

Among the materials used by the researchers, most studies focus on Portland cement of class 42.5, which is partially replaced with fly ash, most commonly Class F; only in study [76] was Class C fly ash also used. In the case of the presented ECC and SCC [77] mixtures, a high-range water reducer was applied. The superplasticizers reported in the majority of the reviewed studies belonged to the group of PCE-based materials.

Another group includes concretes containing metakaolin, as well as the study by Dhakal et al. [76], who applied an experimental design methodology in geopolymer research. When analyzing the independent variables presented in Table 5, the most commonly considered factors in both mortar and concrete studies were the type and amount of admixture, water-to-binder ratio, presence of a superplasticizer, and method of material incorporation. To complement the independent variables, it is important to properly identify the dependent variables, which are most commonly analyzed for mechanical strength (flexural or compressive), workability, plasticity, porosity of the resulting composites, antibacterial properties, and chloride ion permeability. Depending on the intended application of the composites, additional properties, such as photocatalytic behavior, heat of hydration, and material cost, may also be considered. Defining the dependent and independent variables in this manner allows for the selection of computational methods based on the identified priority parameter and the objective of the modification performed. The most frequently cited purpose of such linked data is to obtain a cement composite characterized by the most optimal parameters among those considered in a given experiment. This represents the search for a compromise between cost, mechanical properties, workability, and the retention of exceptional properties, such as antibacterial properties. Most of the studies referred to in this chapter present their results using multivariate analysis of variance (ANOVA) and graphical RSM, where the relationships between the experimental data and the statistically determined approximations can be clearly observed. The identification of the minimum, maximum, and saddle points is a key result of the application of RSM. As outlined by Li et al. [78], the procedure involves four sequential steps: (i) identification of the dependent and independent variables (factors and responses), (ii) selection of the appropriate design strategy to fit the responses using RSM, (iii) use of statistical inference and analysis of variance to confirm the fitted model, and (iv) determination of the optimal experimental conditions. The choice of the mathematical model appropriate to the set of dependent and independent variables presented depends largely on the experience of the analyst. However, it also depends significantly on all input criteria (e.g., the specified objective, parameter priorities, etc.). In each case, it is also important to consider the limitations of the methods that can be applied. For example, when there are a small number of design variables, two-dimensional contour plots work well; however, when more than three independent factors are involved, this method becomes ineffective [72]. The use of statistical methods in experimental work provides an excellent means of presenting laboratory data, both in the form of mathematical equations and graphically through diagrams. This tool effectively supports the process of selecting experimental parameters, leading to more sustainable material production processes while maintaining the principle of technological moderation and ensuring optimal financial management, allowing the production of desired materials/products while respecting cost constraints. Undoubtedly, the use of statistical methods in future research will contribute to a more balanced approach to the management of available raw materials and to maintaining the optimal conditions for processes that allow the production of materials with desirable properties.

**Table 5:** Comparison of dependent and independent data in the design of experiments process for cementitious composites.

Material	Details of the Experiment	Independent Variables	Dependent Variables	Significance	Presentation	Ref.
TiO_2_-SiO_2_/lignin hybrid materials	Cement mortars were prepared using Portland cement CEM I 42.5R, quartz sand, distilled water, and the following admixtures: TiO_2_-SiO_2_, TiO_2_-SiO_2_/lignin (5:1), TiO_2_-SiO_2_/lignin (1:1), TiO_2_-SiO_2_/lignin (1:5), or lignin in amounts of 0.5, 1.0, or 1.5 wt.%	(i) the type of admixture, the quantity of admixture; (ii) the influence of the admixture type	(i) compressive strength, flexural strength, plasticity; (ii) compressive strength, microbial purity CM, microbial purity OD 600, heat of hydration, plasticity, total open porosity	The major objective was to acquire a cement composite with necessary physical and functional characteristics, i.e., optimum strength and flexibility, followed by enhanced antibacterial and structural capabilities	A quadratic D-Optimal design, RSM, ANOVA	[79]
High-performance cement composites (HPCCs) with industrial by-products such as fly ash, silica fume, and colloidal silica	HPCC mix was performed with Portland cement CEM I 42.5N, three types of fine sand, diabase crushed stone, fly ash, silica fume, nanosilica, and different amounts of superplasticizer (0–2.5 wt.% with a 0.5 step)	Fly ash content, nanosilica content, superplasticizer amount, water, and cement amount	Compressive strength (after 28 and 90 days), concrete mix workability parameters (e.g., cone pouring time, cone flow diameter), mix stability in terms of segregation, and raw material cost	Compromise between strength and workability	1st, 2nd, and 3rd order polynomial models, RSM	[80]
Different ZnO oxides	Cement mortars were prepared using Portland cement CEM I 42.5R, quartz sand, distilled water, and the following admixtures: commercially available ZnO oxides (ZnO-CH, ZnO-AA, or ZnO-SA) or synthesized (ZnO-H or ZnO-M) in amounts of 0.1 wt.%	Admixture type (pure cement, ZnO-CH, ZnO-AA, ZnO-SA, ZnO-H, ZnO-M)	Compressive strength; microbial purity; initial setting time; plasticity; cost	The best compressive strength, high microbial purity, and finally, the cost of the ZnO doping agent	I-Optimal model, RSM, ANOVA	[74]
ZnO/lignin hybrid materials	Cement mortars were prepared using Portland cement CEM I 42.5R, quartz sand, distilled water, and the following admixtures: ZnO, lignin or ZnO-lignin hybrid materials in amounts of 0.1 wt.%	Admixture type (pure cement, ZnO, ZnO/lignin (5:1), ZnO/lignin (1:1), ZnO/lignin (1:5))	Compressive strength, microbial purity, porosity, plasticity, and heat of hydration	The best microbial purity, the smallest total pore volume, and satisfactory physical properties	I-Optimal model, RSM, ANOVA	[74]
Deep eutectic solvent with ZnO (DES-ZnO)	Cement mortars were prepared using Portland cement CEM I 42.5R, quartz sand, distilled water, and DES-ZnO admixture in amounts of 0.125, 0.25, or 0.5 wt.%	Concentration of DES-ZnO	Compressive strength (28 days), compressive strength (90 days), microbial purity (CM), microbial purity (OD), porosity, plasticity, heat of hydration	A high level of microbiological purity, the highest values of compressive strength, increased plasticity, and a satisfactory level of porosity	A modified 4^1^ full square I-Optimal design, RSM, ANOVA	[75]
ZnO selection of mixing methods	Cement mortars were prepared using Portland cement CEM I 42.5R, quartz sand, and distilled water in two configurations: (i) with 0.1 wt.% ZnO admixture, and (ii) with 0.1 wt.% ZnO admixture and 0.5 wt.% superplasticizer	The absence/presence of admixture, mixing method, the presence/absence of superplasticizer	Compressive strength, microbial purity, OD method microbial purity, porosity < 2.0 mm, total porosity, plasticity	High microbial purity and satisfactory physical properties, including compressive strength, porosity, and plasticity	The 2FI D-Optimal design, RSM, ANOVA	[72]
CuO	Cement mortars were prepared using Portland cement CEM I 42.5R, quartz sand, distilled water, and CuO admixture in amounts of 0.25, 0.50 or 1.00 wt.%	Concentration of CuO	Compressive strength, microbial purity (CM), microbial purity (OD), porosity (MP), porosity (CT), plasticity	A high level of microbiological purity, with the highest values of compressive strength, increased plasticity, and a satisfactory level of porosity	A randomized quadratic D-Optimal design, RSM, ANOVA	[81]
Various forms of TiO_2_	Cement mortars were prepared using Portland cement CEM I 42.5R, quartz sand, distilled water, and the following admixtures: commercially available TiO_2_ oxides (AN, RdH, TP, P25) or synthesized (TT, TS) in amounts of 0.5, 1.0, or 1.5 wt.%	Concentration and type of TiO_2_ (TT, RdH, TP, TS, P25, AN)	(i) the compressive strength and plasticity; (ii) microbial purity, OD microbial purity, photocatalytic properties, and porosity	(i) the medium value of the concentration of the admixture led to a cement composite with satisfactory compressive strength and plasticity; (ii) maximized strength and plasticity, followed by excellent antimicrobial and photocatalytic properties	(i) An altered 3–6^2^ full factorial D-Optimal design, RSM, ANOVA; (ii) modified 6^1^ full factorial D-Optimal design, RSM, ANOVA	[82]
Hybrid fiber engineered cementitious composite (ECC)	ECC mixes were prepared with cement, class F fly ash, slag, dolomite, dune sand, high-range water reducer, PE and steel fibers	Total cement replacement level, dolomite to binder ratio, slag-to-fly ash ratio, fiber proportions, water-to-binder ratio	Compressive strength, peak compressive strain, elastic modulus, tensile strength, ultimate tensile strain	Total binder content and slag-to-fly ash ratio are key factors influencing the properties of the mixture	Taguchi-Grey relational analysis (GRA) and Taguchi method with utility concept (UC), ANOVA	[83]
Self-compacting cement mortar	Mortars were prepared using two powders (cement CEM I 42.5R and limestone), two PCE-based superplasticizers, sand, and distilled water	Ratios: Water/Cement, Superplasticizer/Powder, Water/Powder, Sand/Mortar; Superplasticiser A or Superplasticiser B	The D-flow and the t-funnel, the compressive strength	Increasing the water/cement ratio improves workability but may negatively affect compressive strength. Superplasticizer A and B affect fluidity and strength differently; a higher sand/mortar ratio may impair self-compacting properties but increase mechanical strength	Central Composite Design (CCD)	[84]
Self-compacting cement mortar	Mortars were prepared using two powders (cement CEM I 42.5R and limestone), two sands (medium sand and fine sand), PCE-based superplasticizer, and distilled water	Ratios: Water/Cement, Superplasticizer/Powder, Water/Powder, Sand/Mortar, Fine Sand/Sand	Workability (the D-flow, the t-funnel), mechanical properties (the tensile and compressive strength)	The correct fine sand/sand ratio allows for an optimal balance between workability and strength. Increasing the water/cement ratio leads to improved workability (higher D-flow), but reduces mechanical strength	CCD	[85]
Self-compacting cement mortar (SCM)	SCM mix were prepared with cement type II, class F fly ash, slag, colloidal nanosilica, superplasticizer, and sand	(i) Water/binder (w/b) ratio, superplasticizer (SP), limestone powder (LSP), binder content (BC); (ii) fly ash (FA), slag (S), nanosilica (NS)	(i) Rheology (slump flow diameter, v-tunnel time); (ii) rheology and compressive strength	Increasing the slag and fly ash content improves the mechanical properties, but may slightly deteriorate the rheology. The optimal ratio of superplasticizer improves mortar flow and mixture stability	Taguchi method, ANOVA	[55]
Concrete containing metakaolin (MK)	Concrete mix was performed with type GU Canadian Portland cement, metakaolin, superplasticizer, natural sand, and stone	Total binder content, percentage of MK, and water-to-binder ratio (W/B)	Rapid chloride permeability test (RCPT), chloride diffusion test, compressive strength, modulus of elasticity, splitting tensile strength, flexural strength, and cost of mixture per cubic meter	To obtain an optimum mixture that achieves a balance between high mechanical/durability properties and lower cost	The developed CCD model, ANOVA, RSM	[86]
Cement mortar with silica fume and nanosilica particles	Mortars mix was prepared with cement CEM I 42.5N, silica fume, nanosilica, natural sand, high-range water reducing admixture, and hydrated lime	Cement mix ingredients and proportions (cement, silica fume (SF), nanosilica (NS))	Compressive strength, flexural strength, splitting strength, absorption, and capillary water	The interactions of CEM × NS and SF × NS indicate that their combination leads to a decrease in water absorption values	A multi-regression analysis using the least-squares method, first and second-order linear models, and ANOVA	[87]
Self-consolidating concrete (SCC) incorporating metakaolin (MK)	SCC mixes were obtained with type GU Canadian Portland cement, metakaolin, slag, class F fly ash, silica fume, high-range water reducer, fine and coarse aggregates	Total binder content, percentage of MK, water-to-binder ratio, and curing conditions	Chloride permeability, fresh and hardened properties of mixes,	To determine the most significant factors affecting the chloride permeability and the expected service life	The Box–Wilson Central Composite Design (CCD) method, ANOVA	[77]
Geopolymer mortar	Geopolymer mortars were prepared with class C and F fly ashes, fine sand, sodium silicate, and sodium hydroxide	Activator to fly ash ratio (AS/FA), fly ash particle size distribution (PSD), silicon and aluminum oxides to calcium ratio ((S + A)/C)	Compressive strength, porosity, microstructure	The compressive strength as a function of (S + A)/C and PSD at respective levels of AS/FA ratio	Second-order regression models, RSM, ANOVA	[76]
High-strength self-consolidating concrete (SCC) incorporating metakaolin (MK)	SCC mixes were obtained with type GU Canadian Portland cement, metakaolin, slag, class F fly ash, silica fume, high-range water reducer, fine and coarse aggregates	Total binder content, percentage of MK in the mixture, and water-to-binder ratio	Flow ability, low segregation factor, superior passing ability, compressive strength	To determine the most significant factors affecting the properties of SCC and the optimum level of each variable	Linear or nonlinear regression analysis, ANOVA	[77]

## 5. Multicharge Cationic Surfactant-Capped Silver Nanoparticles: DoE and RSM in Optimization of Unit Synthetic Process

Capping agents (plant extracts, gums, cationic surfactants, and polymers) play an important role in the synthesis of AgNPs, eliminating agglomeration—due to their high surface energy—of the colloidal particles by surrounding the particles. The application of various capping agents can not only control the nanoparticle size, aggregation, and morphology, but also affect the stability of nanostructures over time. The choice of capping agent for the synthesis of metal nanoparticles is extremely important to achieve the most monodisperse population (narrow size distribution, well-defined shape, and convenient dispersion rate) of nanoparticles, such as AgNPs [88,89,90,91].

Currently, surfactants are continuously designed to achieve products with specific functionalities tailored for specialized applications in fine chemicals, such as catalysts, magnetic surfactants, stabilizing and capping agents, or reactive agents at interfaces [92,93]. Among them, cationic surfactants with multiple head groups are particularly noteworthy for their role in capping and stabilizing activities at the interfaces [10,94,95]. The positively charged head groups of cationic surfactant ligands promote stronger interactions with the nanoparticles, while the hydrophobic chain length provides steric hindrance around the particles, thereby preventing aggregation and enhancing stabilization. Owing to their high hydrophobicity and ability to control morphology, certain surfactants—such as gemini surfactants—have been reported to be more effective shape-directing agents than conventional single-tail, single-head cationic surfactants [96,97]. Numerous studies have emphasized the efficiency of multihead ionic surfactants as stabilizing capping agents in nanoparticle synthesis. M. Pisárčik et al. [98] studied gemini surfactants with biodegradable spacers for AgNP dispersive systems, whereas R.M. Giráldez-Pérez et al. [99] explored gemini surfactant-capped AuNPs (gold nanoparticles) as prospective therapies. A study conducted by J.B. Petersen et al. [100] investigated the solubility properties of initial reagents using Hansen Solubility Parameters for surfactant-capped AgNPs in ink and printing methods. M. Mahmood et al. [101] focused on optimizing the parameters of wet chemical synthesis for quasi-spherical AgNPs stabilized with single- or double-chain surfactants. Only a limited number of studies have exclusively focused on employing DoE and RSM to optimize surfactant-capped nanoparticles. K. Riahi et al. [102] performed surfactant-driven optimization of iron-based nanoparticles, whereas A.L. Nguyen et al. [103] adjusted the surfactant ratio in the precise synthesis of gold nanobipyramids, highlighting the tunable features of plasmon resonances. Consequently, the subsequent paragraphs will elucidate the utilization of DoE in optimizing the synthesis of surfactant-capped AgNPs using multicharge cationic surfactants.

By applying DoE and optimizing the unit synthetic process through RSM, a rational synthetic route for multicharged cationic surfactant-capped AgNPs, which remain colloidally stable over long periods, can be established. Newly synthesized gemini- and dicephalic-type surfactants, containing two or four hydrophilic head groups and two n-dodecyl chains as the hydrophobic moiety, give rise to double-headed and quadruple-headed architectures, respectively. These surfactants were employed as novel capping ligands, and their structural, physicochemical, and synthetic characteristics are summarized in Table 6.

The synthetic route for gemini N,N’-bisdodecyl-N,N’-bis(3-aminopropyl)ethylenediamine dimethanesulfonate (C_12_-GNNH_3_MeSO_3_) and gemini-quadruple N,N’-bisdodecyl-N,N’-bis(N’-(3-aminopropyl)-N’-1,3-diamine)ethylenediamine tetramethanesulfonate (C_12_-GNQNNH_3_MeSO_3_) was performed in a straightforward multi-step procedure (so-called modular synthesis [10]) involving acrylonitrile as the building block and the following synthetic unit operations: Michael’s addition (Step 1), heterogeneous catalytic reduction (Step 2), and neutralization of amine motifs with methanesulfonic acid (Step 3). It should be noted that the first step for the gemini structure included the incorporation of an appropriate linker (ethylenediamine) by alkylation with bromododecane. The repeated purification steps, involving recrystallization and liquid-liquid extraction, enabled the obtainment of the final products (C_12_-GNNH_3_MeSO_3_ and C_12_-GNQNNH_3_MeSO_3_; structures and chemical characterization are listed in Table 6) as well as semi-products with high yield and purity. A detailed description of the synthetic methodology for C_12_-GNNH_3_MeSO_3_ is provided in [106]. Dicephalic N’-(3-aminopropyl)-N’-dodecylpropane-1,3-diamine dimethanesulfonate (C_12_-DNNH_3_MeSO_3_) and dicephalic-quadruple N’-(3-aminopropyl)-N’-[3-[3-[bis(3-aminopropyl)amino] propyl-dodecylamino]propyl] propane-1,3-diamine tetramethanesulfonate (C_12_-DNQNNH_3_MeSO_3_) were synthesized utilizing a similar multi-step procedure, with the exception of the starting material (bromododecane). The detailed synthetic routes and purification steps are described in [107].

**Figure 4:**
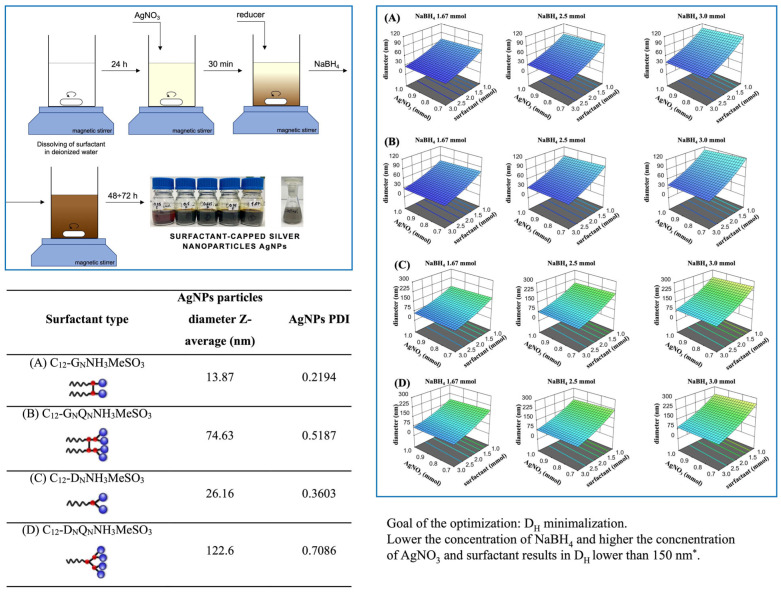
Multicharged cationic surfactants with (A,B) gemini and (C,D) dicephalic architectures as efficient capping agents in silver nanoparticle preparation. (**left bottom**) * The particle size of the resulting AgNPs was measured using dynamic light scattering (DLS, Zetasizer Nano ZS, Malvern Instruments, UK). Simplified procedure for AgNP synthesis (**top right**). Results of the DoE and RSM optimization calculations, represented by a 3-D response surface (**top left**).

The synthesis of AgNPs was performed by incorporating silver salt (AgNO_3_) as a metal precursor, NaBH_4_ as a reducing agent, and C_12_-GNNH_3_MeSO_3_, C_12_-DNNH_3_MeSO_3_, C_12_-GNQNNH3MeSO_3_, or C_12_-DNQNNH_3_MeSO_3_ surfactants. The formation and stability of AgNPs were confirmed by DLS (dynamic light scattering) analyses (PDI (polydispersity index) and D_H_ (hydrodynamic diameter). The results are presented in Figure 4. The key parameters of the AgNP synthesis were as follows: (1) dissolution of a particular surfactant: 24 h, room temperature (RT), speed of mixing 300 rpm (rounds per minute). (2) Formation of sol after the addition of AgNO_3_:30 min, RT, mixing at 300 rpm. (3) Addition of reducing agent: 30 min, T = 4 °C, mixing at 300 rpm. (4) Reduction: 48–72 h, RT, 300 rpm.

A DoE plan, followed by RSM optimization, was employed to identify the optimal synthetic conditions for fabricating AgNPs stabilized by gemini- and dicephilic-type surfactants. This method facilitated a thorough investigation of the essential elements affecting the dimensions and stability of the nanoparticles. These findings underscore the essential influence of surfactant structure on achieving optimal dispersibility and desired physicochemical characteristics. The implemented design, referred to as the I-optimal design, focuses on minimizing the average variance of predictions across the experimental region, emphasizing predictive accuracy rather than estimating parameters. Consequently, specific dependent and independent variables for the synthesis of AgNPs were identified using DoE. Namely, process parameters (A) AgNPs precursor concentration (AgNO_3_), (B) surfactant type (gemini or dicephalic, double or quadruple), (C) particular surfactant concentration (below CMC, within CMC, or above CMC), and (D) reducing agent (NaBH_4_) concentration, respectively, at their distinct leveled values were tested against the response factors, i.e., particle diameter (Y_1_) and their PDI (Y_2_). The statistical analysis conducted through ANOVA tests, along with the assessment of the robustness of the implemented linear multivariate I-optimal model with natural logarithm transformation, followed by multiple regression analysis, facilitated the calculation of the response surfaces for the previously discussed relationship between the independent and dependent variables in the synthesis of AgNPs. The findings of this analysis offer a deeper understanding of the elements affecting the synthesis process. These analyses may inform future research initiatives focused on enhancing the production of AgNPs for diverse applications. The obtained results are shown in Figure 4 as a graphical representation of the computed response surfaces based on multiple regression analysis. The size of AgNPs and PDI was mainly influenced by the type of surfactant and its concentration, with a partially linear positive correlation.

Presentation of the resulting polynomial equations:

ln(diameter) = +4.13 − 0.0370A − 0.4910B +0.2162C − 0.2450D1 − 0.2860D2 R^2^ = 0.8921, Adjusted R^2^ = 0.8694, Predicted R^2^ = 0.7572 (1)

Table 7 defines variables A–D and shows crucial statistical metrics.

To evaluate the quality of the statistical key indicators and the model itself, we compared the exemplary experimental value of the D_H_—13.87 nm (Z-average)—for AgNPs stabilized with the C_12_-GNNH_3_MeSO_3_ gemini surfactant. The validation showed that the D_H_ value predicted by the I-Optimal experimental design under the optimal synthesis conditions for surfactant-capped AgNPs was 17.73 nm, corresponding to an approximate percentage error of 21.5%. This error is consistent with the R^2^ coefficients mentioned above.

The validation indicates that the main limitation of the applied optimization model is the imperfect agreement between predicted and actual values. This discrepancy may be explained by the fact that the mathematical model was treated as linear during computation, whereas the experimental data exhibit a mixed correlation pattern: part of the data follows a linear trend, while the remainder shows a quadratic relationship. Therefore, future refinement of the model—by adding more design points, such as replicate points, center points, and extremum values of the independent variables—could increase its robustness, improve the agreement between the R^2^ coefficients, and ultimately yield a better match between the model and experimental data.

As presented in Figure 4, the chemical structure of the employed capping surfactant, specifically the gemini type with a spacer between hydrophobic chains and fewer polar headgroups, played a key role in stabilizing the formed AgNPs, resulting in stable AgNPs with a D_H_ of 13.87 nm (Z-average). This stability can be attributed to the increased steric hindrance provided by the gemini structure, which effectively prevents the agglomeration of nanoparticles. As shown, the DoE approach coupled with RMS optimization makes it possible to fabricate, in an optimal way, physically stable, long-term, and highly concentrated Ag nanoparticles in aqueous solution using multicharge cationic surfactants branched on the nitrogen atom and containing two (i.e., C_12_-GNNH_3_MeSO_3_ (gemini), C_12_-DNNH_3_MeSO_3_ (dicephalic)) and four (i.e., C_12_-GNQNNH_3_MeSO_3_ (gemini-quadruple), C_12_-DNQNNH_3_MeSO_3_ (dicephalic-quadruple)) cationic hydrophilic groups. We hope that our findings will provide better insights into the custom-designed syntheses of multicharge cationic surfactant-assisted nanoparticles with excellent physical stability for a variety of modern applications.

## 6. Conclusions

Methodologies for process design and optimization are fundamental in the fine and specialty chemical sector, providing powerful tools for the development of innovative products and processes. The integration of experimental design with advanced optimization techniques enables the creation of engineering solutions that are not only economically viable and operationally efficient but also environmentally responsible. The analyzed case studies—rational nanodispersions, ecological nanodetergents, sustainable cementitious composites, and cationic surfactant-capped silver nanoparticles—demonstrate the versatility and broad applicability of these approaches in the design and development of nanostructured materials across interdisciplinary domains.

The systematic application of the Design of Experiments and Response Surface Methodology highlights their critical role in scaling laboratory processes for industrial production, ensuring reproducibility, and optimizing performance parameters. These methods also provide valuable predictive insights that can significantly reduce development time and resource consumption.

*Multicharge cationic surfactant-capped silver nanoparticles synthesis*

The DoE approach combined with RMS optimization enables the optimal fabrication of long-term physically stable and highly concentrated Ag nanoparticles in aqueous solution using multicharged cationic surfactants branched at the nitrogen atom. These surfactants contain either two cationic hydrophilic groups (e.g., C_12_-GNNH_3_MeSO_3_, a gemini surfactant, and C_12_-DNNH_3_MeSO_3_, a dicephilic surfactant) or four such groups (e.g., C_12_-GNQNNH_3_MeSO_3_, a gemini-quadruple surfactant, and C_12_-DNQNNH_3_MeSO_3_, a dicephilic-quadruple surfactant). The primary factors influencing the size of the AgNPs and PDI were the type of surfactant and its concentration, both of which showed a partially linear positive correlation. Moreover, the type of surfactant used during synthesis played a key role. In general, multicharged cationic surfactants with two head groups produced more favorable results than those with four head groups did. The hydrodynamic diameters obtained for the two-headed gemini and dicephalic surfactants were 13.87 and 26.16 nm, respectively, whereas the corresponding four-headed surfactants yielded much larger particles—74.63 and 122.60 nm. In addition, the surfactant architecture influenced both the particle size and the PDI of the resulting AgNPs, with gemini surfactants producing the most desirable nanoparticles.

*Experimental and Process Design: Stages and Objectives—a synthetic overview*

The scientific literature [62,74,108] on DoE/RSM theory and tools consistently emphasizes the importance of a structured and systematic workflow applicable to both academic research and industrial practice. This methodology forms the foundation for the effective implementation of optimization tools in complex technological processes.

Experimental planning begins with a precise definition of the study assumptions, including the research objective and expected outcome. Based on these assumptions, dependent variables representing process responses and independent variables corresponding to controlled input parameters are identified. These variables may be quantitative or qualitative, numerical or categorical, and in the case of numerical variables, either continuous or discrete. The appropriate selection of process parameters, along with their ranges and levels, enables a reliable assessment of their influence on key process responses, such as yield, product quality, and functional properties.

The next step involves defining the optimization criterion, which determines whether the process response should reach a predetermined value or range, be minimized, or maximized. This criterion guides the subsequent statistical analysis and interpretation of results.

An algorithm describing the technological process or unit operation is then developed, taking into account the relationships among variables and the structure of the experimental design. Based on this algorithm, a series of experiments is conducted according to the established experimental design matrix, encompassing the planned combinations of independent variables. This approach ensures the collection of a sufficiently comprehensive dataset for quantitative evaluation of the effects of input factors on the process response.

The final stage includes statistical analysis of the experimental results and formulation of conclusions. Appropriate statistical methods, most notably analysis of variance (ANOVA), were applied to assess the significance of individual factors and the adequacy of the optimization model. The outcomes of this analysis provide a basis for verifying model assumptions and proposing potential modifications that can lead to further process improvements.

*Key limitations of the DoE/RSM approaches*

Although DoE followed by RSM are powerful and relatively flexible tools for process and product design and optimization, they exhibit several limitations. The main issue underlying DoE/RSM is that their foundations rely primarily on polynomial assumptions, which typically do not exceed first- or second-order terms when approximating the relationship between the dependent and independent variables. As a result, polynomial-based models may fail to accurately describe complex or highly nonlinear systems.

This limitation leads to another drawback: response surfaces generated using this approach are usually valid only within the narrow experimental range of the studies conducted. Consequently, extrapolating the model outside the experimental domain may yield unreliable outcomes. Finally, response surface methodology is often sensitive to experimental error and noise inherent in the measurements, which can ultimately lead to suboptimal or misleading optimization results [109,110,111,112].

*A forward-looking perspective*

Moreover, several contributions have revealed that the integration of artificial neural network (ANN) models can, in certain cases, provide superior predictive accuracy and process-response mapping compared with classical RSM alone. This is especially evident in highly nonlinear systems or in multicomponent formulations typical of FCs and SCs, where ANN-based optimization captures complex variable interactions more effectively. The complementary use of ANN with DoE-generated datasets offers an advanced hybrid strategy that not only improves optimization efficiency but also reduces analytical workload, accelerates decision-making, and lowers overall development costs [41,44].

Looking forward, the integration of data-driven modeling, machine learning, and real-time process monitoring holds great potential for further enhancing process efficiency and innovation. This study establishes a strong foundation for future advancements in sustainable nanotechnology and reinforces the importance of methodological rigor in driving the next generation of high-performance, customized nanoproducts in the chemical processing industry. The examples of nanodispersions presented in this study emphasize the versatility of DoE and optimization approaches, particularly in their application to improve the formulation and performance of nanoproducts in various chemical processes.

## Figures and Tables

**Table 2:** Parameters of the ecological nanodetergent production process proposed by the Design of Experiments for Response Surface Methodology optimization.

Process Parameters	Values
Constant parameters	
Oil-PEG 8 content (%wt.)	38.5
Biosolvent content (%wt.)	45.0
Water content (%wt.)	14.0
Temperature (°C) ^a^	25.0
Independent variables	
Type of surfactant	APG; AAS
Concentration of surfactant (mol)	0.050; 0.075; 0.100
Pressure Homogenization (MPa)	0.1; 100; 150
Dependent variables	Goal of optimization
Particles diameter (nm)	100–500
PDI	<1
TSI	<5 (The value of the parameter should not change by more than 5 units within a 90-day interval)

^a^ formulation inlet temperature to the HPH process; APG—alkyl polyglucoside surfactant; AAS—amino acid surfactant; PDI—polydispersity index; TSI—turbiscan stability index.

**Table 3:** Summary of physicochemical characterization of selected ecological nanodetergents.

	NE No. 1 [64]	NE No. 2 [64]	NE No. 3 [65]	NE No. 4 [59]	NE No. 5 [59]
Ecological surfactant	^a^ APG C_8_-C_10_	SCG	SCMT	^a^ APG C_8_-C_10_	^a^ APG C_8_-C_10_
Concentration	0.1	0.05	0.05	0.1	0.1
Green solvents	EL	EL	EL	LIM	MMB
	UCO-PEG 8				
	Water				
Particle diameter—DH (µm)	0.175 ± 0.05	0.186 ± 0.04	0.508 ± 0.11	0.270 ± 0.42	399 ± 0.62
Polydispersity index—PDI	0.030	0.037	0.044	0.024	0.024
Turbiscan stability index—TSI	2.14 ± 0.05	0.06 ± 0.04	1.51 ± 0.05	1.76 ± 0.04	3.54 ± 0.05

^a^—C_8_–C_10_ refers to carbon atoms in the alkyl chain.

**Table 4:** Comparison of the wetting properties of w/o nanoemulsions stabilized by APG and AAS on sensitive surfaces and paint coatings [59,64,65].

Type of Surface	*W_A_*	*W_S_*
	mJ/m^2^	
Paint without additives	84.5 ± 2.0	−62.9 ± 2.8
Paint with nitrocellulose	78.4 ± 1.1	−62.3 ± 18.0
Paint with bitumen	82.4 ± 2.8	−58.8 ± 17.1
Glass	134.1 ± 2.3	−11.5 ± 2.4
Aluminum	88.6 ± 3.2	−20.6 ± 2.8
Marble	125.0 ± 5.2	−42.2 ± 4.7
Stone	103.4 ± 4.7	−57.0 ± 5.3

*W_A_* (work of adhesion), *W_S_* (spreading work).

**Table 6:** Comparison of dependent and independent data in the design of experiments process in the research of cementitious composites.

Structure, Name and Abbreviation	^1^H NMR ^a^, DMSO-d_6_ d (ppm)	^13^C NMR ^a^, DMSO-d_6_ d (ppm)	ESI-MS ^b^ (M^+^)	Elementary Analyses ^c^ (Theoretical Values)			MP ^d^ (°C)	CMC ^e^ (mM)	T_K_ ^f^ (°C)
				C (%)	H (%)	N (%)			
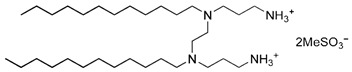 N,N’-bisdodecyl-N,N’-bis(3-aminopropyl)ethylenediamine dimethanesulfonate C_12_-G_N_NH_3_MeSO_3_ (gemini)	0.89–0.92 [t, 6H, CH_3_(CH_2_)_10_CH_2_N<]; 1.25–1.50 [m, 40H, CH_3_(CH_2_)_10_CH_2_N<]; 2.10–2.14 [m, 4H, >NCH_2_CH_2_CH_2_NH_3_^+^]; 2.36–2.47 [m, 18H, N(CH_2_-)_3_]; 2.85 [s, 6H, CH_3_SO_3_^−^]; 3.32 [m, 4H, >NCH_2_CH_2_CH_2_NH_3_^+^]; 6.92–7.14 [m, 6H, -NCH_2_CH_2_CH_2_NH_3_^+^]	14 [-(CH_2_)_10_CH_3_]; 20–30 [-(CH_2_)_10_CH_3_, >NCH_2_CH_2_-]; 38 [-CH_2_NH_3_^+^]; 41 [CH_3_SO_3_^−^]; 50–55 [N(CH_2_-)_3_]	607.55 (M^+^)	57.96 (58.07)	11.11 (11.20)	10.03 (7.97)	168.0–169.5	2.1 (2.0 ^g^)	<0
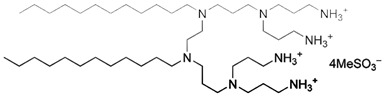 N,N’-bisdodecyl-N,N’-bis(N’-(3-aminopropyl)-N’-1,3-diamine)ethylenediamine tetramethanesulfonate C_12_-G_N_Q_N_NH_3_MeSO_3_ (gemini-quadruple)	0.89–0.92 [t, 6H, CH_3_(CH_2_)_10_CH_2_N<]; 1.25–1.50 [m, 44H, CH_3_(CH_2_)_10_CH_2_N<, >NCH_2_CH_2_CH_2_N<]; 2.13–2.15 [m, 8H, >NCH_2_CH_2_CH_2_NH_3_^+^]; 2.35–2.47 [m, 36H, N(CH_2_-)_3_]; 2.85 [s, 12H, CH_3_SO_3_^−^]; 3.33 [m, 8H, >NCH_2_CH_2_CH_2_NH_3_^+^]; 6.90–7.10 [m, 12H, -NCH_2_CH_2_CH_2_NH_3_^+^]	14 [-(CH_2_)_10_CH_3_]; 20–30 [-(CH_2_)_10_CH_3_, >NCH_2_CH_2_-]; 38 [-CH_2_NH_3_^+^]; 41 [CH_3_SO_3_^−^]; 52–55 [N(CH_2_-)_3_]	442.39 (M^2+^)	51.45 (51.30)	10.38 (10.25)	9.87 (9.97)	174.5–175.0	2.88	<0
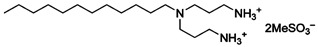 N’-(3-aminopropyl)-N’-dodecylpropane-1,3-diamine dimethanesulfonate C_12_-D_N_NH_3_MeSO_3_ (dicephalic)	0.92–0.94 [t, 3H, CH_3_(CH_2_)_10_CH_2_N<]; 1.26–1.40 [m, 20H, CH_3_(CH_2_)_10_CH_2_N<]; 2.10–2.14 [m, 8H, >NCH_2_CH_2_CH_2_NH_3_^+^]; 2.35–2.37 [m, 8H, N(CH_2_-)_3_]; 2.84 [s, 6H, CH_3_SO_3_^−^]; 3.33 [m, 8H, >NCH_2_CH_2_CH_2_NH_3_^+^]; 6.95–7.12 [m, 6H, -NCH_2_CH_2_CH_2_NH_3_^+^]	14 [-(CH_2_)_10_CH_3_]; 20–30 [-(CH_2_)_10_CH_3_, >NCH_2_CH_2_-]; 38 [-CH_2_NH_3_^+^]; 41 [CH_3_SO_3_^−^]; 52–55 [N(CH_2_-)_3_]	396.33 (M^+^)	48.61 (48.85)	9.91 (10.06)	8.58 (8.55)	152–153	30.0	<0
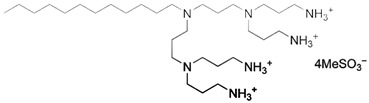 N’-(3-aminopropyl)-N’-[3-[3-[bis(3-aminopropyl)amino]propyl-dodecylamino]propyl]propane-1,3-diamine tetramethanesulfonate C_12_-D_N_Q_N_NH_3_MeSO_3_ (dicephalic-quadruple)	0.91–0.93 [t, 3H, CH_3_(CH_2_)_10_CH_2_N<]; 1.25–1.50 [m, 24H, CH_3_(CH_2_)_10_CH_2_N<, >NCH_2_CH_2_CH_2_N<]; 2.11–2.14 [m, 8H, >NCH_2_CH_2_CH_2_NH_3_^+^]; 2.35–2.38 [m, 36H, N(CH_2_-)_3_]; 2.83 [s, 12H, CH_3_SO_3_^−^]; 3.33 [m, 8H, >NCH_2_CH_2_CH_2_NH_3_^+^]; 6.98–7.15 [m, 12H, -NCH_2_CH_2_CH_2_NH_3_^+^]	14 [-(CH_2_)_10_CH_3_]; 20–30 [-(CH_2_)_10_CH_3_, >NCH_2_CH_2_-]; 38 [-CH_2_NH_3_^+^]; 41 [CH_3_SO_3_^−^]; 51–55 [N(CH_2_-)_3_]	360.78 (M^2+^)	44.79 (44.76)	9.59 (9.41)	10.61 (10.75)	155–168	35.0	<0

^a^ All spectra were recorded on a Bruker AMX-500 spectrometer (Bruker, Billerica, MA, USA) in DMSO-d_6_ at concentrations between 5 and 10 mg/mL. ^b^ Determined using electrospray ionization mass spectroscopy (ESI-MS) (micrOTOF-Q instrument; Bruker Daltonics, Bremen, Germany, calibrated with the Tunemix^TM^ mixture; the spectra were analyzed using the DataAnalysis 3.4 software (Bruker Daltonics, Germany) with a resolution of at least 5 ppm). ^c^ Performed uwing Vario EL cube (Elementar, Langenselbold, Germany), calibrated on acetanilide. ^d^ Determined using the Boetius apparatus (Carl Zeiss, Oberkochen, Germany). ^e^ From conductometric measurements. ^f^ Determined as in ref. [104]. ^g^ From ref. [105].

**Table 7:** Comparison of dependent and independent data in the design of experiments process for cementitious composites.

Independent Variables			Levels			
A: AgNO_3_ (mmol)			L1	L2	L3	
B: type of surfactant			L1	L2	L3	L4
C: surfactant concentration			<CMC	CMC	>CMC	
D: NaBH_4_ (mmol)			L1	L2	L3	
Dependent variables			Goal			
Y_1_ = average particle diameter			Minimization			
Y_2_ = PDI			Minimization			
Source	Sum of Squares	df	Mean Square	F-value	*p*-value	
Model	9.34	5	1.87	6.39	0.0008	
A-AgNO_3_	0.0216	1	0.0216	0.0737	0.7885	
B-Surfactant	4.28	1	4.28	14.64	**^a^ 0.0009**	
C-NaBH_4_	0.7853	1	0.7853	2.69	0.1154	
D-Surfactant type	4.05	2	2.02	6.93	**^a^ 0.0046**	
Residual	6.43	22	0.2923			
Lack of Fit	6.43	17	0.3783			
Pure Error	0.0000	5	0.0000			
Cor Total	15.77	27				

^a^ bolded values represent statistical significance.

## Data Availability

No new data were created or analyzed during this study. Data sharing is not applicable to this article.

## Abbreviations

The following abbreviations are used in this manuscript:

6R	Refuse, Reduce, Reuse, Recover, Recycle, Rethink
AAS	Amino acid surfactant
AgNPs	Silver nanopartic
ANN	Artificial neural network
ANOVA	Analysis of variance
APG	Alkylpolyglucoside
API	Active pharmaceutical ingredients
BBD	Box-Behnken design
CCD	Central composite design
CCs	Commodity chemicals
CMC	Critical micelle concentration
D_H_	Hydrodynamic diameter
DLS	Dynamic light scattering
DoE	Design of experiments
EL	Ethyl lactate
FCs	Fine chemicals
FD	Factorial Design
GMP	Good manufacturing practice
HPH	High-pressure homogenization
LIM	D-limonen
MP	Melting point
ND	Nanodispersions
PDI	Polydispersity Index
QbD	Quality-by-design
REACH	Registration, Evaluation, Authorization and Restriction of Chemicals
RO	Rapeseed oil
RSM	Response surface methodology
SCG	Sodium cocoyl glycinate
SCs	Specialty chemicals
SMCT	Sodium methyl cocoyl taurate
UCO	Used cooking oil
w/o	Water-in-oil

## References

[1] Cybulski A., Moulijn J.A., Sharma M.M., Sheldon R.A., Science B.V. (2001). Fine Chemicals Manufacture: Technology and Engineering.

[2] Pollak P. (2011). Fine chemicals. The Industry and the Business.

[3] Tickner J., Geiser K., Baima S. (2021). Transitioning the Chemical Industry: The Case for Addressing the Climate, Toxics, and Plastics Crises. Environ. Sci. Policy Sustain. Dev..

[4] Stokes R., Kipping M., Kurosawa T., Westney E. (2022). Chemical industries: Changes in products, processes, actors. The Oxford Handbook of Industry Dynamics.

[5] Lantos J., Kumar N., Saha B. (2024). A Comprehensive Review of Fine Chemical Production Using Metal-Modified and Acidic Microporous and Mesoporous Catalytic Materials. Catalysts.

[6] Ciriminna R., Della Pina C., Luque R., Pagliaro M. (2024). Reshoring Fine Chemical and Pharmaceutical Productions. Org. Process Res. Dev..

[7] Mrowiec-Białoń J., Ciemięga A., Maresz K., Szymańska K., Pudło W., Jarzębski A.B. (2018). Review on hierarchically microstructured monolithic reactors for high yield continuous production of fine chemicals. Chem. Proc. Eng..

[8] Szczęsna W., Ciejka J., Szyk-Warszyńska L., Jarek E., Wilk K.A., Warszyński P. (2022). Customizing polyelectrolytes through hydrophobic grafting. Adv. Colloid Interface Sci..

[9] Nagtode V.C., Cardoza C., Yasin H.K.A., Mali S.J., Srushti M., Tambe S.M., Roy P., Singh K., Goel A., Amin P.D. (2023). Green Surfactants (Biosurfactants): A Petroleum-Free Substitute for Sustainability—Comparison, Applications, Market, and Future Prospects. ACS Omega.

[10] Lamch Ł., Szczęsna W., Balicki S.J., Bartman M., Szyk-Warszyńska L., Warszyński P., Wilk K.A. (2023). Multiheaded cationic surfactants with dedicated functionalities: Design, synthetic strategies, self-assembly and performance. Molecules.

[11] Shaikh M.A.N., Nawat T. (2024). Highly Efficient Cationic Surfactant Functionalized Alginate Hydrogel for Perfluorooctanoic Acid Adsorption: Optimization through Response Surface Methodology and Performance Evaluation for Aqueous Media. ACS EST. Water.

[12] Qin S., Omolabake S., Diaby A., Li J., González L.D., Holland C.M., Zavala V.M., Stahl S.S., Van Lehn R.C. (2024). Identifying Green Solvent Mixtures for Bioproduct Separation Using Bayesian Experimental Design. ACS Sustain. Chem. Eng..

[13] Bensebaa F., Bensebaa F. (2013). Chapter 5—Clean Energy. Interface Science and Technology.

[14] Panizza M., Butterworth-Heinemann, Martínez-Huitle C.A., Rodrigo M.A., Scialdone O. (2018). Chapter 13—Fine Chemical Industry, Pulp and Paper Industry, Petrochemical Industry and Pharmaceutical Industry. Electrochemical Water and Wastewater Treatment.

[15] Ciriminna R., Della Pina C., Luque R., Pagliaro M. (2025). The Fine Chemical Industry, 2000−2024. Org. Process Res. Dev..

[16] Ledakowicz S., Antecka A., Gluszcz P., Klepacz-Smolka A., Pietrzyk D., Szelag R., Slezak R., Daroch M. (2024). From 3G biofuels to high-value-added bioproducts. Chem. Proc. Eng. New Front..

[17] Wang S., Li X., Ma R., Song G. (2025). Catalytic hydrogenolysis of lignin into serviceable products. Acc. Chem. Res..

[18] Zhang Y., Gao F., Fu M.-L. (2018). Composite of Au-Pd nanoalloys/reduced graphene oxide toward catalytic selective organic transformation to fine chemicals. Chem. Phys. Lett..

[19] Landge S., Melvin C., Pence A., Török B. (2025). Microwave-Assisted Synthesis of Fine Chemicals—Triazoles. Encyclopedia of Green Chemistry.

[20] Thompson B., Machas M., Nielsen D.R. (2015). Creating pathways towards aromatic building blocks and fine chemicals. Curr. Opin. Biotechnol..

[21] Ash M., Ash M., Ash I. (2009). Specialty Chemicals Source Book.

[22] Zhou J., Metivier P. (2023). Science—An important lever to tackle sustainability in the specialty chemical industry. Nat. Sci. Rev..

[23] Lorenzett A.K.P., Babinski T.P., de Lima V.A., Mainardes R.M. (2025). Optimization of Eudragit RS100 Nanocapsule Formulation for Encapsulating Perillyl Alcohol and Temozolomide Using Design of Experiments. ACS Nanosci. Au.

[24] Manda A., Komati S.K., Nariyam S.M., Annapurna S.C.V., Senadi G.C., Maruthapillai A., Bandichhor R. (2024). Olaparib Process Development Employing Quality by Design (QbD) Principles. ACS Omega.

[25] Chen H., Li Q., Deng S. (2024). Fast QoI-Oriented Bayesian Experimental Design with Unified Neural Response Surfaces for Kinetic Uncertainty Reduction. Energy Fuels.

[26] Kiraly L.M., Friedler F., Szoboszlai L. (1989). Optimal design of multi-purpose batch chemical plants. Comput. Chem. Eng..

[27] Jaryal V.B., Villa A., Gupta N. (2023). Metal-Free Carbon-Based Nanomaterials: Insights from Synthesis to Applications in Sustainable Catalysis. ACS Sustain. Chem. Eng..

[28] Rezaee M., Feyzi F., Javanshir S. (2025). Application of Response Surface Methodology for Selective Extraction of Lithium Using a Hydrophobic Deep Eutectic Solvent. Ind. Eng. Chem. Res..

[29] Tan J.D., Ramalingam B., Chellappan V., Gupta N.K., Dillard L., Khan S.A., Galvin C., Hippalgaonkar K. (2024). Generative Design and Experimental Validation of Non-Fullerene Acceptors for Photovoltaics. ACS Energy Lett..

[30] Castilla-Caballero D., Medina-Guerrero A., Hernandez-Ramirez A., Vazquez-Rodriguez S., Colina-Márquez J., Martínez F.M., Barraza-Burgos J., Roa-Espinosa A., Gunasekaran S. (2025). Use of a 416B-type central-hybrid experimental design to evaluate the synthesis conditions of TiO_2_/biochar composites on the solid-state photocatalytic degradation of polypropylene-plastic films. Appl. Catal. A Gen..

[31] Melikhova E.Y., Smith D.A., Moseley J.D. (2024). Application of Experimental Design (DoE) to Improve a Very Dilute Workup Procedure. Org. Process Res. Dev..

[32] Zhang Y., An M., Han B., Wang J., Wang Y. (2025). Multi-parameter mix proportion design and optimization of manufactured sand concrete based on RSM. Structures.

[33] Jakowluk W., Świercz M. (2020). Application-oriented experiment design for model predictive control. Bull. Pol. Acad. Sci. Tech. Sci..

[34] Zou Y., Ma X., Yang Y., Li S. (2025). An overview of chemical process operation-optimization under complex operating conditions. Digit. Chem. Eng..

[35] Web of Science. http://www.webofscience.com.

[36] Pandey S., Kumar S. (2020). Reactive extraction of gallic acid from aqueous solution with Tri-n-octylamine in oleyl alcohol: Equilibrium, Thermodynamics and optimization using RSM-rCCD. Sep. Purif. Technol..

[37] Rahimi S., Mikani M. (2019). Lycopene green ultrasound-assisted extraction using edible oil accompany with response surface methodology (RSM) optimization performance: Application in tomato processing wastes. Microchem. J..

[38] Hashemifesharaki R., Xanthakis E., Altintas Z., Guo Y., Gharibzahedi S.M.T. (2020). Microwave-assisted extraction of polysaccharides from the marshmallow roots: Optimization, purification, structure, and bioactivity. Carbohydr. Polym..

[39] Bai H., Tian J., Talifu D., Okitsu K., Abulizi A. (2022). Process optimization of esterification for deacidification in waste cooking oil: RSM approach and for biodiesel production assisted with ultrasonic and solvent. Fuel.

[40] Lu J., Shi Y., Huang K., Liu Y., Yuan S., Yang X., Xu Y., Sun X., Wu T. (2025). Improved Synthesis for the 4-Pyridone Intermediate of Baloxavir Marboxil: Elimination of Polar Aprotic Solvents and Optimization Through Design of Experiments (DoE). Org. Process Res. Dev..

[41] Gammoudi N., Mabrouk M., Bouhemda T., Nagaz K., Ferchichi A. (2021). Modeling and optimization of capsaicin extraction from *Capsicum annuum* L. using response surface methodology (RSM), artificial neural network (ANN), and Simulink simulation. Ind. Crops Prod..

[42] Mellinas A.C., Jiménez A., Garrigós M.C. (2020). Optimization of microwave-assisted extraction of cocoa bean shell waste and evaluation of its antioxidant, physicochemical and functional properties. LWT.

[43] Wang H., Li L., Yang F., Li D., Wu S., Yan W., Sun G., Tan J., Li Y., Yang H. (2025). Process development and optimization of apalutamide synthesis aided by the Design of Experiments (DoE). Tetrahedron.

[44] Anand A., Mahata C., Moholkar V.S. (2024). Biohydrogen synthesis from food waste hydrolysate: Optimization using statistical design of experiments (DoE) and artificial neural network (ANN). Biomass Bioenergy.

[45] Avşar D., Özcan Z.I., Iyisan B. (2025). Optimizing the synthesis of bovine serum albumin nanoparticles using full factorial design of experiments. Mater. Res. Express.

[46] Onder A.C., Tomak A., Karakus C.O. (2023). Optimizing the dispersion of calcium phosphate nanoparticles for cellular studies using statistical design of experiments. Ceram. Int..

[47] Triboandas H., Bezerra M., Almeida J., de Castro M., Santos B.A.M.C., Schlindwein W. (2024). Optimizing extrusion processes and understanding conformational changes in itraconazole amorphous solid dispersions using in-line UV-Vis spectroscopy and QbD principles. Int. J. Pharm. X.

[48] Sanmartín P., Cappitelli F., Mitchell R. (2014). Current Methods of Graffiti Removal: A Review. Constr. Build. Mater..

[49] Melquiades F.L., Appoloni C.S., Andrello A.C., Spagnuolo E. (2019). Non-destructive analytical techniques for the evaluation of cleaning and protection processes on white marble surfaces. J. Cult. Herit..

[50] Gomes V., Dionísio A., Pozo-Antonio J.S. (2017). Conservation strategies against graffiti vandalism on Cultural Heritage stones:Protective coatings and cleaning methods. Prog. Org. Coat..

[51] Baglioni M., Raudino M., Berti D., Keiderling U., Bordes R., Holmberg K., Baglioni P. (2014). Nanostructured fluids from degradable nonionic surfactants for the cleaning of works of art from polymer contaminants. Soft Matter.

[52] Baglioni M., Poggi G., Benavides Y.J., Martínez Camacho F., Giorgi R., Baglioni P. (2018). Nanostructured fluids for the removal of graffiti—A survey on 17 commercial spray-can paints. J. Cult. Herit..

[53] Baglioni M., Poggi G., Giorgi R., Rivella P., Ogura T., Baglioni P. (2021). Selective removal of over-paintings from “Street Art” using an environmentally friendly nanostructured fluid loaded in highly retentive hydrogels. J. Colloid Interface Sci..

[54] Chen S., Hu S., Song J., Chen T., Xing H., Kong C. (2026). Hydrogels for the cleaning of cultural heritage: A review of mechanisms, applications, and future perspectives. J. Cult. Herit..

[55] Jalal M., Teimortashlu E., Grasley Z. (2019). Performance-Based Design and Optimization of Rheological and Strength Properties of Self-Compacting Cement Composite Incorporating Micro/Nano Admixtures. Compos. Part B Eng..

[56] Baglioni M., Giorgi R., Berti D., Baglioni P. (2012). Smart Cleaning of Cultural Heritage: A New Challenge for Soft Nanoscience. Nanoscale.

[57] Chelazzi D., Bordes R., Casini A., Mastrangelo R., Holmberg K., Baglioni P. (2025). New perspectives on green sustainable wet cleaning systems for art conservation. Soft Matter.

[58] Bartman M., Balicki S., Hołysz L., Wilk K.A. (2024). Benefits of using nonionic saccharide surfactant-based detergents for nanostructured fluids as stubborn graffiti paint remover. J. Surfactants Deterg..

[59] Carretti E., Dei L., Baglioni P. (2003). Solubilization of Acrylic and Vinyl Polymers in Nanocontainer Solutions. Appl. Microemulsions Micelles Cult. Herit. Conserv. Langmuir.

[60] Lettieri M., Masieri M., Pipoli M., Morelli A., Frigione M. (2019). Anti-Graffiti Behavior of Oleo/Hydrophobic Nano-Filled Coatings Applied on Natural Stone Materials. Coatings.

[61] Anastas P.T., Zimmerman J.B. (2019). The Periodic Table of the Elements of Green and Sustainable Chemistry. Green. Chem..

[62] Bartman M., Balicki S., Hołysz L., Wilk K.A. (2023). Graffiti coating eco-remover developed for sensitive surfaces by using an optimized high-pressure homogenization process. Colloids Surf. A Physicochem. Eng. Asp..

[63] Bartman M., Balicki S., Wilk K.A. (2021). Formulation of environmentally safe graffiti remover containing esterified plant oils and sugar surfactant. Molecules.

[64] Bartman M., Hołysz L., Balicki S., Szczęsna-Górniak W., Wilk K.A. (2024). Wettability of Graffiti Coatings by Green Nanostructured Fluids. ChemPhysChem.

[65] Bartman M., Balicki S., Hołysz L., Wilk K.A. (2023). Surface properties of graffiti coatings on sensitive surfaces concerning their removal with formulations based on the amino-acid type surfactants. Molecules.

[66] Voicu G., Tiuca G.-A., Badanoiu A.-I., Holban A.-M. (2022). Nano and mesoscopic SiO_2_ and ZnO powders to modulate hydration, hardening and antibacterial properties of Portland cements. J. Build. Eng..

[67] Chen J., Kou S., Poon C. (2012). Hydration and properties of nano-TiO_2_ blended cement composites. Cem. Concr. Compos..

[68] Amor F., Baudys M., Racova Z., Scheinherrová L., Ingrisova L., Hajek P. (2022). Contribution of TiO_2_ and ZnO nanoparticles to the hydration of Portland cement and photocatalytic properties of High Performance Concrete. Case Stud. Constr. Mater..

[69] Najafi Kani E., Rafiean A.H., Alishah A., Hojjati Astani S., Ghaffar S.H. (2021). The effects of nano-Fe_2_O_3_ on the mechanical, physical and microstructure of cementitious composites. Constr. Build. Mater..

[70] Sikora P., Augustyniak A., Cendrowski K., Nawrotek P., Mijowska E. (2018). Antimicrobial Activity of Al_2_O_3_, CuO, Fe_3_O_4_, and ZnO Nanoparticles in Scope of Their Further Application in Cement-Based Building Materials. Nanomaterials.

[71] Horszczaruk E., Łukowski P., Seul C. (2020). Influence of dispersing method on the quality of nano-admixtures homogenization in cement matrix. Materials.

[72] Klapiszewska I., Ławniczak Ł., Balicki S., Gapiński B., Wieczorowski M., Wilk K.A., Jesionowski T., Klapiszewski Ł., Ślosarczyk A. (2023). Influence of zinc oxide particles dispersion on the functional and antimicrobial properties of cementitious composites. J. Mater. Res. Technol..

[73] Ślosarczyk A., Klapiszewska I., Jędrzejczak P., Klapiszewski Ł., Jesionowski T. (2020). Biopolymer-based hybrids as effective admixtures for cement composites. Polymers.

[74] Klapiszewska I., Balicki S., Wilk K.A., Klapiszewski Ł., Ślosarczyk A. (2023). Statistical approach to the production of cement composites doped with ZnO and ZnO-based materials. Physicochem. Probl. Miner. Process..

[75] Klapiszewska I., Latos P., Parus A., Balicki S., Lodowski P., Wilk K.A., Jesionowski T., Chrobok A., Klapiszewski Ł., Ślosarczyk A. (2023). New insights into sustainable cementitious composites doped with a hybrid system based on zinc oxide and a designable deep eutectic solvent. J. Mater. Res. Technol..

[76] Dhakal M., Kupwade-Patil K., Allouche E.N., la Baume Johnson C.C., Ham K., Kriven W.M., Wang J., Zhou Y., Gyekenyesi A.L. (2014). Optimization and characterization of geopolymer mortars using response surface methodology. Developments in Strategic Materials and Computational Design IV, The American Ceramic Society.

[77] Abouhussien A.A., Hassan A.A. (2014). Application of statistical analysis for mixture design of high-strength self-consolidating concrete containing metakaolin. J. Mater. Civ. Eng..

[78] Li Z., Lu D., Gao X. (2021). Optimization of mixture proportions by statistical experimental design using response surface method—A review. J. Build. Eng..

[79] Jędrzejczak P., Parus A., Mildner M., Klapiszewska I., Balicki S., Kołodziejczak-Radzimska A., Siwińska-Ciesielczyk K., Fiala L., Wilk K.A., Černý R. (2024). The novel incorporation of lignin-based systems for the preparation of antimicrobial cement composites. Int. J. Biol. Macromol..

[80] Sahmenko G., Rucevskis S., Lusis V., Spure L., Korjakins A., Annamaneni K.K., Bajare D. (2024). Elaboration mix design methodology for obtaining defined properties of cement composite with fly ash, silica fume and colloidal silica. Mech. Compos. Mater..

[81] Ślosarczyk A., Klapiszewska I., Parus A., Balicki S., Kornaus K., Gapiński B., Wieczorowski M., Wilk K.A., Jesionowski T., Klapiszewski Ł. (2023). Antimicrobial action and chemical and physical properties of CuO-doped engineered cementitious composites. Sci. Rep..

[82] Jędrzejczak P., Parus A., Balicki S., Kornaus K., Janczarek M., Wilk K.A., Jesionowski T., Ślosarczyk A., Klapiszewski Ł. (2023). The influence of various forms of titanium dioxide on the performance of resultant cement composites with photocatalytic and antibacterial functions. Mater. Res. Bull..

[83] Rawat S., Zhang Y.X., Lee C.K. (2022). Multi-response optimization of hybrid fibre engineered cementitious composite using Grey-Taguchi method and utility concept. Constr. Build. Mater..

[84] Maia L. (2022). Experimental dataset from a central composite design with two qualitative independent variables to develop high strength mortars with self-compacting properties. Data Brief..

[85] Maia L. (2021). Experimental dataset from a central composite design to develop mortars with self-compacting properties and high early age strength. Data Brief..

[86] Al-alaily H.S., Hassan A.A.A. (2016). Refined statistical modeling for chloride permaeability and strength of concrete containing metakaolin. Constr. Build. Mater..

[87] Hameed M.F.A.E., Ghazy M.F., Elaty M.A.A.A. (2016). Cement mortar with nanosilica: Experiments with mixture design method. ACI Mater. J..

[88] Restrepo C.V., Villa C.C. (2021). Synthesis of silver nanoparticles, influence of capping agents, and dependence on size and shape: A review. Environ. Nanotechnol. Monit. Manag..

[89] Shrestha S., Wang B., Dutta P. (2020). Nanoparticle processing: Understanding and controlling aggregation. Adv. Colloid Interface Sci..

[90] Cardellini A., Alberghini M., Govind Rajan A., Misra R.P., Blankschtein D., Asinari P. (2019). Multi-scale approach for modeling stability, aggregation, and network formation of nanoparticles suspended in aqueous solutions. Nanoscale.

[91] Heuer-Jungemann A., Feliu N., Bakaimi I., Hamaly M., Alkilany A., Chakraborty I., Masood A., Casula M.F., Kostopoulou A., Oh E. (2019). The role of ligands in the chemical synthesis and applications of inorganic nanoparticles. Chem. Rev..

[92] Shehzad F., Hussain S.M.S., Adewunmi A.A., Mahboob A., Murtaza M., Kamal M.S. (2021). Magnetic surfactants: A review of recent progress in synthesis and applications. Adv. Colloid Interface Sci..

[93] Polarz S., Kunkel M., Donner A., Schlötter M. (2018). Added-value surfactants. Chem. Eur. J..

[94] Warszyński P., Szyk-Warszyńska L., Wilk K.A., Lamch Ł. (2022). Adsorption of cationic multicharged surfactants at liquid/gas interface. Curr. Opin. Colloid Interface Sci..

[95] Ahmady R., Hosseinzadeh P., Solouk A., Akbari S., Szulc A.M., Brycki B.E. (2022). Cationic gemini surfactant properties, its potential as a promising bioapplication candidate, and strategies for improving its biocompatibility: A review. Adv. Colloid Interface Sci..

[96] Brycki B., Szulc A., Babkova M. (2021). Synthesis of silver nanoparticles with gemini surfactants as efficient capping and stabilizing agents. Appl. Sci..

[97] Song T., Gao F., Guo S., Zhang Y., Li S., You H., Du Y. (2021). A review of the role and mechanism of surfactants in the morphology control of metal nanoparticles. Nanoscale.

[98] Pisárčik M., Záteková M., Oláhová K., Lukáč M., Jampílek J., Bilková A., Bilka F., Devínsky F., Bezina M., Brezani V. (2024). Role of gemini surfactants with biodegradable spacer as efficient capping agents of silver nanoparticles. J. Drug. Deliv. Sci. Technol..

[99] Giráldez-Pérez R.M., Grueso E., Lhamyani S., Perez-Tejeda P., Gentile A.-M., Kuliszewska E., Roman-Perez J., El Bekay R. (2020). miR-21/Gemini surfactant-capped gold nanoparticles as potential therapeutic complexes: Synthesis, characterization and in vivo nanotoxicity probes. J. Mol. Liq..

[100] Petersen J.B., Meruga J., Randle J.S., Cross W.M., Kellar J.J. (2014). Hansen Solubility Parameters of Surfactant-Capped Silver Nanoparticles for Ink and Printing Technologies. Langmuir.

[101] Mahmood M., Abid M., Nazar M.F., Zafar M.N., Raza M.A., Ashfaq M., Khan A.M., Sumrra S.H., Zubair M. (2020). The wet chemical synthesis of surfactant-capped quasi-spherical silver nanoparticles with enhanced antibacterial activity. Mater. Adv..

[102] Riahi K., Dirba I., Ablets Y., Filatova A., Sultana S.N., Adabifiroozjaei E., Molina-Luna L., Number U.A., Gutfleisch O. (2023). Surfactant-driven optimization of iron-based nanoparticle synthesis: A study on magnetic hyperthermia and endothelial cell uptake. Nanoscale Adv..

[103] Nguyen A.L., Griffin Q.J., Wang A., Zou S., Jing H. (2025). Optimization of the Surfactant Ratio in the Formation of Penta-Twinned Seeds for Precision Synthesis of Gold Nanobipyramids with Tunable Plasmon Resonances. J. Phys. Chem. C.

[104] Wang Y., Zhang Y., Liu X., Wang J., Wei L., Feng Y. (2014). Effect of a Hydrophilic Head Group on Krafft Temperature, Surface Activities and Rheological Behaviors of Erucyl Amidobetaines. J. Surfactants Deterg..

[105] Sokołowski A., Wilk K.A., Komorek U., Rutkowski B., Syper L. (2002). Aggregation properties of cationic gemini surfactants in aqueous solution. Physicochem. Probl. Miner. Process..

[106] Wilk K.A., Syper L., Domagalska B.W., Komorek U., Maliszewska I., Gancarz R. (2002). Aldonamide-type gemini surfactants: Synthesis, structural analysis, and biological properties. J. Surfactants Deterg..

[107] Wilk K.A., Syper L., Burczyk B., Sokołowski A., Domagalska B.W. (2002). Synthesis and Surface Properties of New Dicephalic Saccharide-Derived Surfactants. J. Surfactants Deterg..

[108] Balicki S. (2021). Unit processes optimization in the organic technology. Przem. Chem..

[109] Baş D., Boyacı I.H. (2007). Modeling and optimization I: Usability of response surface methodology. J. Food Eng..

[110] Pietraszek J., Radek N., Goroshko A.V. (2020). Challenges for the DOE methodology related to the introduction of Industry 4.0. Prod. Eng. Arch..

[111] Deaconu S., Coleman H.W. (2000). Limitations of Statistical Design of Experiments Approaches in Engineering Testing. J. Fluids Eng..

[112] Wu C.F.J., Hamada M.S. (2021). Experiments: Planning, Analysis and Optimization.

